# Barefoot Trees for Bees: Nesting Site Characteristics of the Ground‐Nesting Bee *Andrena vaga* in an Urban Environment

**DOI:** 10.1002/ece3.72327

**Published:** 2025-10-18

**Authors:** Hanna Gardein, Tim Diekötter, Elke Bloem, Henri Greil

**Affiliations:** ^1^ Institute for Bee Protection Julius Kühn Institute (JKI) – Federal Research Centre for Cultivated Plants Braunschweig Germany; ^2^ Department of Landscape Ecology, Institute for Natural Resource Conservation Christian‐Albrecht‐University Kiel Germany; ^3^ Institute for Crop and Soil Science Julius Kühn Institute (JKI) – Federal Research Centre for Cultivated Plants Braunschweig Germany

**Keywords:** green infrastructure, habitat selection, insect loss, soil‐nesting bee, solitary wild bee, urban biodiversity

## Abstract

Ground‐nesting bees constitute the majority of all wild bee species, but are critically endangered. Despite their crucial role in pollination and soil health, knowledge about the nesting requirements of ground‐nesting bees is lacking, with most nesting site descriptions being vague and unsupported by empirical data. In our study, we conducted extensive measurements of relevant soil parameters at 27 nesting sites of the oligolectic mining bee species *Andrena vaga* within the city of Braunschweig, Germany, and compared them to uncolonized control areas, where no bee nests were found. *Andrena vaga* nested in (loamy) sand and sandy loam, and nesting sites were preferably located near trees under canopy cover. We identified the proportion of bare ground, soil temperature, and soil hardness, as well as the water content, to be the main factors distinguishing nesting sites from uncolonized areas. Artificially created bare ground plots located within control areas did not provoke colonization of *Andrena vaga* , assumingly due to social aspects, but were colonized by other bee orders such as *Lasioglossum*. The causal relationships between bee nest presence and soil characteristics are unclear, but likely bidirectional: ground‐nesting bees tend to prefer drier soils with sparse vegetation, while potentially increasing proportions of bare ground and enhancing soil drainage, thus acting as environmental engineers. Our results allow conclusions about how public places like parks, cemeteries, or roadsides can be managed to provide suitable nesting sites for ground‐nesting bees.

## Graphical abstract

*Andrena vaga* nesting sites were preferably located under canopy cover. The soils of the nesting sites were warmer and drier and showed less penetration resistance and vegetation cover compared to uncolonized control areas. Experimental bare ground plots located within control areas did not provoke colonization of *Andrena vaga* , assumingly due to social aspects, but of small bee species such as *Lasioglossum* spp.

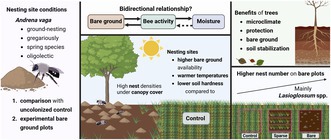

## 1 Introduction

Ground‐nesting bees constitute the majority of all wild bee species (Danforth et al. 2019; Harmon‐Threatt 2020), and provide essential ecosystem services for both natural and agricultural ecosystems: Next to their well‐recognized role in pollination, they significantly contribute to soil health (Christmann 2022; Tschanz et al. 2025). Similar to earthworms, ground‐nesting bees rework the soil structure and create macropores (Tschanz et al. 2023), thus potentially increasing gas exchange and water drainage (Tschanz et al. 2025). Further, they can enrich the soil with nutrients due to their brood cell construction, e.g., with leaves, and the mass provisioning of pollen and nectar (Tschanz et al. 2025). However, this important functional group is more strongly declining compared to cavity nesting bees (Graham et al. 2024), as they are particularly vulnerable to lacking nesting opportunities, nest damage by human activities, and accumulation of harmful substances in the soil (Harmon‐Threatt 2020). New insights into their nesting requirements may help to develop measures that contribute to their conservation.

Especially in urban contexts, ideal nesting sites for ground‐nesting bees are thought to be rare due to impervious surfaces, intensive management practices of green spaces, or compaction (Geslin et al. 2016; Pereira et al. 2021; Liang et al. 2023). Nevertheless, cities can exhibit high habitat heterogeneity and a moderate degree of anthropogenic soil disturbances that lead to beneficial habitat conditions (Wenzel et al. 2020; Prendergast et al. 2022). These soil disturbances are typically less destructive than intensive management practices in agricultural landscapes, which can destroy brood cells of ground‐nesting bees (Tschanz et al. 2024). With a high availability, diversity, and temporal continuity of floral resources and nesting opportunities, cities can harbor high species numbers and abundances of wild bees (e.g., Theodorou et al. 2020). Particularly, allotments, parks, or cemeteries serve as refuges for wild bees due to their long‐term habitat stability (Baldock et al. 2019; Weber et al. 2023).

Bottom‐up forces, such as food or nesting site availability, can have stronger impacts on the population dynamics of wild bees than top‐down forces (e.g., parasitism) (Steffan‐Dewenter and Schiele 2008). Often, bee species have specific preferences regarding their nesting site (Maher et al. 2019), spending considerable time in finding the optimal location to ensure ideal conditions for offspring development and hibernation and thus for their survival (Brockmann 1979; Danforth et al. 2019). As the female bee dies after brood cell completion and cannot take care of its offspring afterward, selecting an ideal nest location is essential to safeguard the vulnerable and immobile larval stage against adverse impacts (Antoine and Forrest 2021). While the importance of floral resources is well studied, nesting site requirements are mainly investigated for cavity‐nesting bees (Potts et al. 2005; Harmon‐Threatt 2020; Antoine and Forrest 2021). These rare autecological studies often rely only on a single nesting site and provide rather vague descriptions such as ‘sandy soil’, ‘steep slope’, or ‘sparse vegetation’ (Harmon‐Threatt 2020). Precise measurements of nesting site parameters are crucial for targeted conservation measures and the prediction of focal species occurrences, but also for laboratory experiments and pollinator management in food production (Cane 1997; Harmon‐Threatt 2020). Comparisons with uncolonized areas and manipulative approaches can help to identify key parameters for nest site selection mechanisms (Roulston and Goodell 2011; Antoine and Forrest 2021).

The aim of this study was to determine the nesting site characteristics of the ground‐nesting mining bee *Andrena vaga* (Hymenoptera: Andrenidae), a solitary but gregariously nesting wild bee, forming large nest aggregations (Westrich 2019). We investigated 27 nesting sites in and around Braunschweig, a city in Central Germany. Over three years, we mapped the total number of nests and the spatial distribution of nest densities within nesting sites. Conditions within the nesting sites were compared with adjacent uncolonized controls, analyzing soil samples and vegetation characteristics. We investigated both the soil characteristics that determine where this bee species nests, as well as the bees' impact on the soil properties, which might be particularly high for gregarious nesting bees (Tschanz et al. 2025). As bare ground availability was reported to be the main factor influencing the nesting site selection of *A. vaga* (Falk 1991; Bischoff 2003; Fellendorf et al. 2004) and other ground‐nesting bee species (e.g., Ballare et al. 2019; Fountain et al. 2023; Antoine et al. 2025), we tested whether *A. vaga* would colonize the control areas with artificially increased bare ground availability. For this purpose, experimental plots with sparse and without any vegetation cover were set up on previously uncolonized areas next to the nesting sites.

## 2 Material and Methods

### 2.1 Biology of *Andrena vaga*

The grey‐backed mining‐bee *Andrena vaga* Panzer, 1799 is common in central Europe (Danforth et al. 2019). Still, it shows a scattered distribution, as it requires specific habitat characteristics regarding both nesting and food resources: *Andrena vaga* prefers ‘sandy’ soils (Bischoff 2003; Rezkova et al. 2012) and is specialized on willows as a pollen source (*Salix* spp.) (Westrich and Schmidt 1987; Bischoff et al. 2003). Thus, it often colonizes riverine habitats like river basins (Rezkova et al. 2012) or dams (Vleugel 1947; Fellendorf et al. 2004). *Andrena vaga* is a univoltine early spring species with a flight period between mid‐March and early May (Bischoff et al. 2003; Westrich 2019). This solitary bee nests gregariously, forming large aggregations that occasionally exceed several tens of thousands of nests, but also occurs in much smaller nest numbers (Fellendorf et al. 2004; Exeler et al. 2008; Westrich 2019). These nest aggregations can persist for several decades (e.g., 60 years; Ulrich 1956). The nest exhibits one vertical main burrow with one or two lateral tunnels reaching a depth of 25–60 cm (Westrich 2019), while nests were mainly found within 30–40 cm (personal observation). The bees hibernate as adults that are fully developed by August (Fellendorf et al. 2004). Since other bee species sometimes nest within *A. vaga* nesting sites (Hallmen 1990), the nesting requirements of *A. vaga* appear to overlap with those of other ground‐nesting bees, suggesting that insights gained from *A. vaga* may be applicable to other species.

### 2.2 Study Area and Study Sites

The study was conducted in Braunschweig (Lower Saxony, Germany), a city with approximately 250.000 citizens and an area of 193 km^2^ (Stadt Braunschweig 2023). Three rivers (Oker, Schunter & Wabe) flow through the city, resulting in predominantly sandy soils due to fluvial deposits, as well as dune formation (Stadt Braunschweig 2021). Mainly within the city limits, 27 nesting sites were studied (Figure S1). Nesting site locations were identified through a combination of the authors' prior knowledge and citizen science contributions following a public call in local newspapers. Site selection was constrained by the limited knowledge of nesting locations, as well as the need for accessibility and cooperation with landowners. From the known nesting sites (*n* = 35), we selected sites representing a broad spatial distribution across the city, covering varying degrees of urbanization and different directions from the city center. The selected nesting sites were located on different types of urban green spaces: public parks (*n* = 7), pathways on extensive grassland (*n* = 4), premises of public institutions (*n* = 3), playgrounds (*n* = 3), cemeteries (*n* = 3), roadside verges (*n* = 3), private front yards (*n* = 3), and one sports field.

### 2.3 Aggregation Size and Nest Densities

**FIGURE 1:**
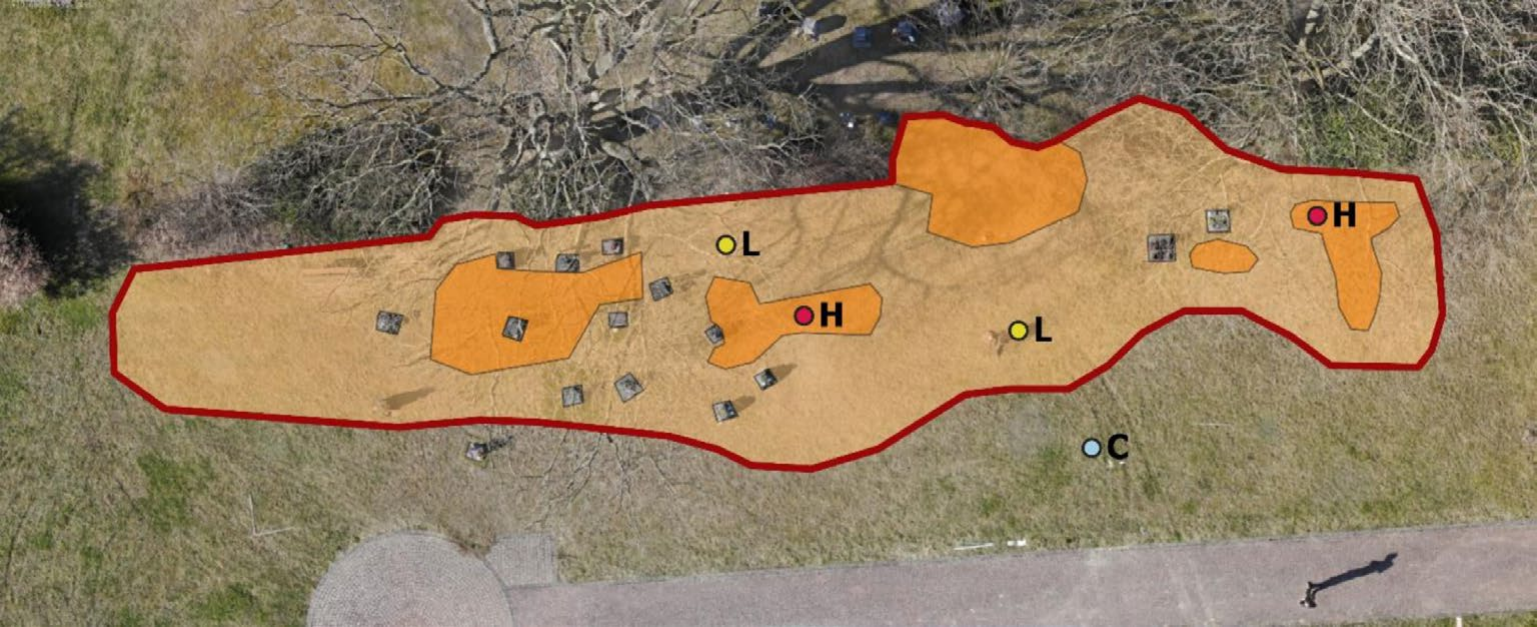
Nesting site on a cemetery (study site 1) with the total aggregation area indicated by the dark red outer line and areas with the high (HND‐area) or low (LND‐area) nest density in dark or light orange, respectively. The blue point (C) represents the control area; yellow (L = ‘low’) and red (H = ‘high’) points show locations of nest‐count quadrats in 2023.

For all 27 nesting sites, the total number of nests (‘aggregation size’) was estimated over a three‐year period (2022–2024), using four nest‐count quadrats (1 m^2^) per study site, as described in Neyns et al. (2025). The colonized area (‘aggregation area’) was determined in situ and mapped on drone images (Figure 1), which were recorded from a standardized height of 30 m in March 2022. The images were taken in spring when trees adjacent to the nesting sites had no leaves, allowing us to map the locations of nests under canopy cover. Additionally, the area with a comparatively high nest density within each nesting site was mapped (hereafter: ‘HND‐area’), and its proportion of the total aggregation area was calculated using QGIS version 3.14 (QGIS Development Team 2020). The counterpart, i.e., the area with a comparatively lower nest density, is referred to as ‘LND‐area’ in the following. Due to overall differences in nesting densities across sites, the definitions of HND‐ and LND‐areas were set individually for each nesting site and were based on pronounced, noticeable disparities in nest densities. Some nesting sites were quite densely colonized throughout the whole aggregation area, with nest densities in HND‐areas being only two times higher as compared to LND‐areas, while some sites showed distinct drops of nest densities, with nest densities in HND‐areas up to seven times higher than in LND‐areas.

### 2.4 Nesting Site Conditions

To determine the abiotic factors that *A. vaga* prefers for nesting, soil characteristics were recorded within each nesting site and a corresponding uncolonized control. The control plot (1 m^2^) was located approximately 2–5 m away from the edge of the respective nesting site in 2021 (Figure 1), outside of the colonized area but close enough to detect fine‐scale habitat differences that could explain nest absence. Control areas were chosen based on the assumption that they were generally suitable for nesting of *A. vaga* , and showed no obvious disparities to the colonized area, e.g., not fully shaded or on stony ground. General site conditions in the control plot were chosen to match those of the sample plot within the nesting site, with identical exposure and no or only minor variations in slope.

#### 2.4.1 Soil Samples and Soil Compaction

In May and June 2021, three soil samples were taken within a 1 m^2^ plot, each in an HND‐area and the corresponding control using a drill core (Pürckhauer gouge auger). Soil cores had a length of 60 cm and a diameter of 28 mm, and were subdivided into the depths of 0–15, 15–30, 30–45, and 45–60 cm. The three samples per depth were lumped together for either the HND‐area or the control, respectively. Combined samples were analyzed regarding proportion of soil skeleton, dry matter, total carbon and nitrogen, as well as texture, pH, conductivity, potassium, and phosphorus. The full method description can be found in the supplement.

In short, soil samples were air‐dried at room temperature and sieved through a 2 mm sieve. The proportion of the soil skeleton was determined by weighing out the fraction > 2 mm and the sieved fraction. Soil texture was determined partly according to DIN ISO 11277, but by using Atterberg cylinders for sedimentation and determination of the clay content. Soil pH was measured by a Seven Multi pH meter and conductivity at 21.7°C by a Seven Multi conductivity meter (both Mettler Toledo, Germany). The proportion of soil dry matter was determined by drying 10 g of fresh soil at 105°C for 24 h and by determining the difference in weight. From this dried soil, total carbon (C) and nitrogen (N) were measured via dry combustion in a C:N analyzer (Vario Max Cube, Elementar, Germany). Available soil potassium (K_2_O) and phosphorus (P_2_O_5_) were determined in a calcium acetate lactate (CAL) extract according to Schüller (1969) and were analyzed by Inductively Coupled Plasma—Optical Emission Spectrometry (ThermoFisher Scientific iCAP 6000 series, Cambridge, UK). All results from the soil samples, as well as geolocations of the nesting sites, are published as a data repository (Gardein et al. 2025).

The soil penetration resistance (in the following ‘hardness’) was determined within a 1 m^2^ plot using a penetrologger (RP0615; Eijkelkamp, Netherlands) until a depth of 60 cm, four times each within the HND‐area and the control. This was done in March 2022, when bees began to excavate their nests. In case of very strong soil compaction or a stone encounter, the penetration was repeated in a slightly different location. Still, most of the penetrations did not reach 60 cm (only 50% of the penetrations), and only one‐fifth reached 30 cm, so missing values were substituted with the preceding measurement. We then calculated the mean soil hardness for the four depth classes (0–15, 15–30, 30–45, 45–60 cm). Soil type classification followed the BÜK200 data (BGR 2013).

#### 2.4.2 Temperature, Slope, and Exposition

To measure soil temperature, four thermochron iButtons DS1921G‐F5# (Maxim Integrated, USA) were buried per study site in May/June 2021: two within the nesting site and two in the control. They were located in the same 1 m^2^ plot where soil samples were taken, but next to the drill core penetration spots. Following our personal observation that the majority of brood cells of *A. vaga* is situated at approximately 40 cm, we chose 20 cm and 40 cm as measurement depths. The iButtons were wrapped in pieces of rubber gloves and tape to waterproof them. They measured soil temperature (°C) every 90 min for one year. The mean temperature per depth was calculated for two crucial time periods: the time during development (15.06.2021–31.08.2021 = ‘soil temperature 1’) and during hibernation (01.09.2021–15.03.2022 = ‘soil temperature 2’). During the flight period between March and May 2022, two data loggers per site were installed on the soil surface within the HND‐areas and control, respectively, recording soil surface temperature (°C) and light intensity (Lux) every hour. To identify differences regarding the time of day, we calculated both the mean daily temperature (8 am until 8 pm) as well as the mean morning temperature (8 am until 12 am). The slope of nesting sites was measured with a slope triangle in 2022 and 2023 at one point in the HND‐area and in the control area, respectively. If the nesting site was located on a slope, the exposure was determined.

#### 2.4.3 Vegetation and Canopy Cover

Vegetation characteristics were surveyed in April 2022 and 2023, halfway through the activity period of *A. vaga* . Surveys took place after a rain event to reduce the amount of heaped soil covering the vegetation, two times each in HND‐ and LND‐areas, and one time in the control. Within the HND‐areas, we chose two locations with the highest nest density; within the LND‐areas the two locations were chosen randomly. In each case, the proportions of vegetation, bare ground, moss, and litter were estimated within one square meter (in %). As we suspected to overestimate the proportion of bare ground during the activity of *A. vaga* due to deposited soil, we additionally determined the vegetation characteristics before the bees' activity in March 2023 on four representative plots that were known to be within the HND‐ and LND‐areas of 2022, respectively.

While mapping nest densities on drone images, we observed that nesting sites were preferably located adjacent to or under trees. Therefore, we also mapped the canopy cover over nesting sites with the help of the drone images (Figure S2). On the basis of visible branches, the canopy cover was estimated and the proportion of the whole aggregation area as well as of the HND‐areas under the canopy was calculated for three years (2022–2024) in QGIS version 3.14 (QGIS Development Team 2020). The tree species under which nests were found were identified, and their diameter in breast height (DBH) was determined.

### 2.5 Experimental Bare Ground Plots

To test whether *A. vaga* would nest within the control areas if the vegetation cover was sparser or even absent, two experimental plots (each 1 m^2^) with different proportions of bare ground were established in 2024 next to a subset of thirteen nesting sites (Figure S3). These plots were located where no nests were found in the previous three years. At the beginning of March, on one plot, the vegetation was completely removed with a spade (‘bare’), on the second plot, only the dead vegetation, litter, and moss were removed with a rake (‘sparse’), and one plot was left unmanipulated (‘control’). In two sampling rounds (mid and end of April 2024), nests of ground‐nesting bees were counted on the plots by thoroughly searching for excavated soil and nest entrances from different angles, as well as within the vegetation, for nest‐building or provisioning bees. This was consistently done by the same person to minimize the bias from different evaluations. Nests of other bee species that did not have the characteristic size and appearance of *A. vaga* nests (e.g., not with buried nest entrances as done by *A. vaga* (Figure S4); Malyshev 1926; Vleugel 1947) were categorized into ‘small’ and ‘large’, depending on their size (1–3 mm tunnel diameter or larger). If possible, nest‐building bees emerging from nests were caught for identification in the laboratory. Additionally, the proportions of vegetation, moss, litter, and bare ground were estimated in both sampling rounds (in % per plot).

### 2.6 Statistical Analysis

All statistical analyses were performed in R version 4.3.0 (R Core Team 2023). Figures were created in R with the package ggplot2 (Wickham 2016) and ggeffects (Lüdecke 2018). All explanatory variables were tested for collinearity using Pearson's correlation coefficient (*r*), visualized with *corrplot.mixed* from the corrplot package (Wei and Simko 2021). We chose a conservative threshold, considering variables with *r* > 0.6 as strongly correlated, and subsequently excluded them from simultaneous inclusion in the models. Since this applied to several parameter pairs (Figure S7), multiple models based on different combinations of variables were calculated (Table S1). The proportions of bare ground and vegetation cover were consistently correlated. Therefore, we retained only the proportion of bare ground as an explanatory variable in all models. For models with multiple random factors, we applied generalized linear mixed effects models, which were fitted using Template Model Builder (TMB) from the glmmTMB package (Brooks et al. 2017). We used stepwise model simplification via the Akaike Information Criterion (AIC). Model fit was assessed using *simulateResiduals* from the DHARMa package (Hartig 2020). Additionally, the marginal and conditional *R* ^2^ values were calculated for the best‐fitting model with *r.squaredGLMM* (Nakagawa and Schielzeth 2013; Johnson 2014) from the MuMIn package (Burnham and Anderson 2002). If singularity was detected, random factors were interpreted using *VarCorr* (Bates et al. 2015). The best model was chosen using AIC, *R* ^2^ and residual fit. All statistical models (full and best models) are listed in Table S1.

### 2.7 Conditions Within Nesting Sites

For the vegetation characteristics within nesting sites, we compared the proportions of bare ground, litter, and moss in April 2022 and 2023 in the HND‐areas with the LND‐areas. We used a glmmTMB with the plot type (HND vs. LND) as a dependent variable, the vegetation characteristics as the explanatory variable, and the site ID and year as random factors (family = ‘binomial’; m1). Additionally, we implemented the same model for the data from March 2023 (without ‘year’ as a random factor; m2) to compare the effects between the time before and during the bees' activity.

If a tree was present in or near the nesting sites (all but three study sites), we analyzed whether *A. vaga* preferred to nest under the tree (Figure S5). Thus, we calculated the expected proportion of the HND‐area under canopy cover and compared it with the observed values (Figure S6). The expected proportion was defined as the proportion of canopy cover of the whole aggregation area, expecting a similar distribution for the HND‐areas. First, we tested the normality of the differences between observed and expected values using the *shapiro.test* function. Based on this result, we applied a paired Wilcoxon test to assess whether the values of each study site differed significantly (m3a). As the *p*‐value was statistically significant, we used a one‐sided Wilcoxon test (alternative = ‘greater’) to test whether the observed proportion of HND‐area was higher than the expected (m3b).

Additionally, we implemented a glmmTMB to see whether the proportion under canopy cover depends on the nest density. We calculated the area (%) under canopy cover and without canopy cover for the HND‐ and LND‐area, respectively. In this model, the area was the dependent variable, and the interaction between nest density (type = ‘HND’ or ‘LND’) and canopy cover (tree = ‘tree’ or ‘no tree’) as well as their main effects were included as explanatory variables. Both the study site and year were used as random factors. Because of real zeros within the data, we used a Tweedie distribution (m4).

### 2.8 Comparison With Control

To identify the key parameter that defines whether bees nest in an area or not, two models were calculated with the plot type (nesting site = 1 and control = 0) as the dependent variable, using a binomial distribution. The first model included all soil parameters with below‐ground data separated in the four depths (pH, conductivity, C:N, soil hardness, soil temperature 1/2, potassium, phosphorus, and proportions of sand, soil skeleton, and dry matter) as fixed effects, as well as their interaction factors with the depth, using study site as a random factor (m5). Post hoc pairwise comparisons of the proportion of dry matter between depth levels were conducted within each plot using the *emmeans_test* function with Bonferroni correction to adjust *p*‐values. The second model (m6) included soil surface parameters (bare ground, moss, litter, soil surface temperature, and light intensity during morning/day). To allow a statistical comparison between the nesting sites and the control, the mean proportions of all four vegetation recordings within the nesting sites, and then the mean of both years for both plot types, were calculated. All explanatory variables were scaled.

### 2.9 Soil Parameters

To understand the relationships between the soil parameters and the influence of the bees' nesting activity (i.e., the presence of bee nests) on them, we calculated two Structural Equation Models (SEMs) implemented by the package piecewiseSEM (Lefcheck 2016). The first SEM was used to describe the bees' impact on the soil properties, namely the proportions of bare ground, dry matter, nitrogen, carbon, potassium, and phosphorus, as well as the soil hardness (based on Tschanz et al. 2025). The second SEM was implemented to describe the interrelations between the parameters identified with models m5 and m6 as potentially relevant for the bees' nesting site selection. We first defined individual models (LMERs) for each response variable while avoiding feedback loops. Here, we used the mean values over all four depths, as it resulted in the best model fit, and incorporated multicollinearity into the SEMs if not already included within the individual models. If necessary, we used a log‐ or sqrt‐transformation for the response variable. We performed a multi‐step model selection, removing weak paths, checking the model fit using Fisher's C test and AIC. Full and best SEMs, as well as model results (Tables S2, S3), can be found in the supplement.

### 2.10 Experimental Bare Ground Plots

Due to a data deficiency for *A. vaga* nests and nests of other larger bee species on the experimental bare ground plots (Table S10), we only statistically analyzed the nest number of the other, smaller bee species found nesting on the plots. The models were fitted using the Tweedie error distribution due to the large number of zeros in the data set. The study site and sampling round were used as random factors. At first, we tested whether the number of nests found on the plots depends on the type (‘bare’, ‘sparse’, or ‘control’; m7). In a second model, the surface characteristics on the plots (proportions of bare ground, moss, and litter) were used as explanatory variables to identify the most important parameter for colonization (m8).

## 3 Results

### 3.1 Aggregation Size and Nest Densities

Over the period of three years, the aggregation sizes varied significantly, while the pattern was specific for each nesting site (Figure S8). Most aggregation sizes showed fluctuations, with a deviation in the second year (Table S4). Six aggregations constantly grew, while seven declined, with two aggregations showing a drastic drop in 2024. The mean aggregation size was 13,027.80 ± 1980.95 nests (230–98,942 nests) and the mean aggregation area was 265.04 ± 23.68 m^2^ (34–978 m^2^; Table S4). The mean proportion of HND‐area was 38.07% ± 1.91% (7.55%–80.5%; Table S4). The highest nest density within HND‐areas was 280 nests per m^2^, the lowest 12 nests, and the mean 105.81 nests per m^2^ (Table S5). We found other bee species, such as *Colletes cunicularius* , *Halictus rubicundus* , and *Lasioglossum* spp., nesting within the nesting sites, albeit in relatively small numbers and rather at the edges of the nesting sites.

### 3.2 Conditions Within Nesting Sites

**FIGURE 2:**
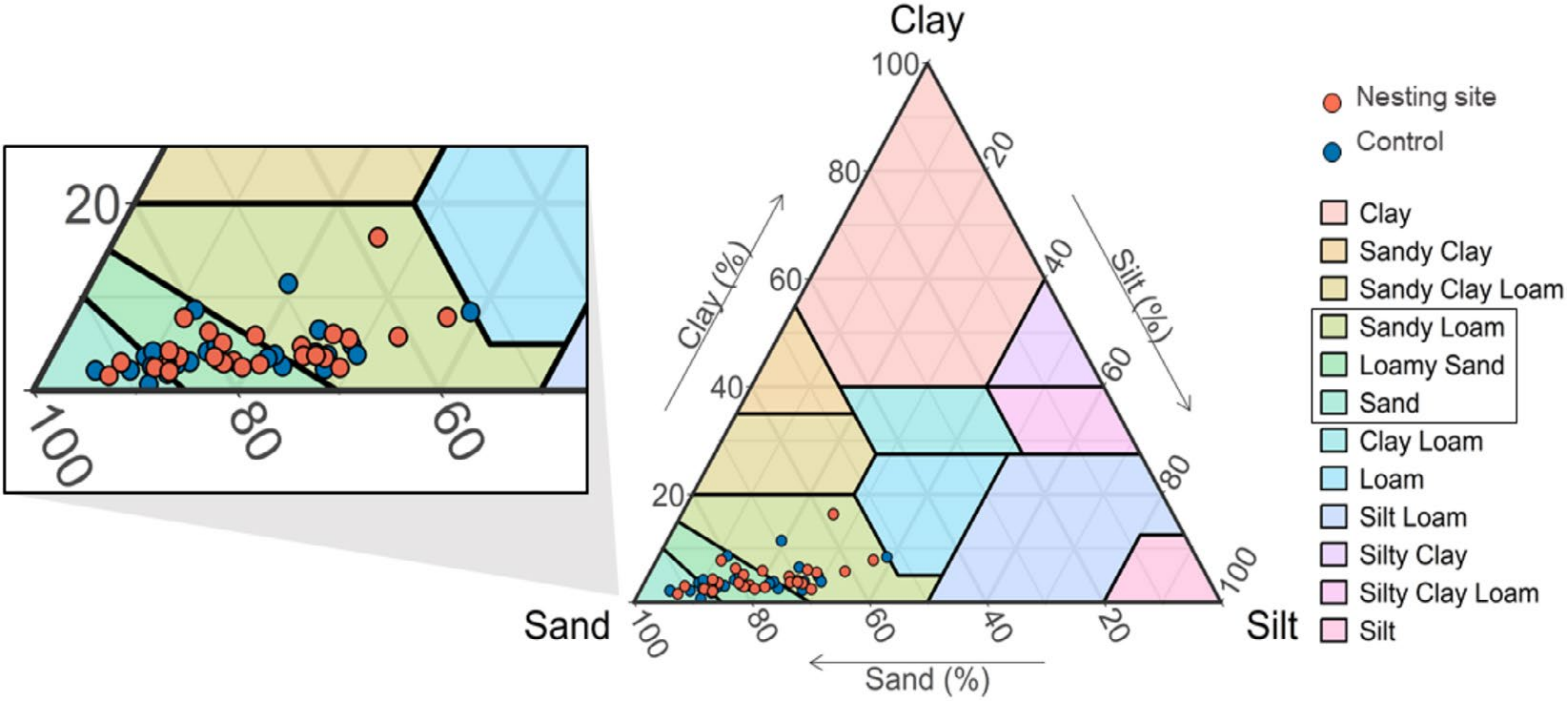
Soil texture in the upper 15 cm, both within the nesting sites (red dots) and the controls (blue dots).

*Andrena vaga* colonized flat as well as inclined areas (14.6% ± 16.58%, 0%–63.75%) with south‐eastern to western exposures (Table S6). The bees nested in sandy soils (sand, loamy sand, and sandy loam; see Figure 2), with a mean sand proportion of 77.2% ± 9.32% (55.3%–93.7%), silt proportion of 18.4% ± 8.29% (4.32%–36.9%), and clay proportion of 4.4% ± 2.29% (1.52%–16.4%). The mean proportion of soil skeleton was 8.5% ± 0.78% (0.03%–43.66%). The soil within the nesting sites had a mean pH value of 7.24 ± 0.08 (5.53–8.91) and conductivity of 55.45 ± 2.82 μS/cm (16.27–124.7 μS/cm), with a mean dry matter proportion of 94.38% ± 0.18% (89.5%–98.7%). The mean proportion of total carbon within the dry matter was 0.86% ± 0.05% (0.09%–2.49%) and of nitrogen 0.06% ± 0.004% (0.01%–0.21%), resulting in a C:N ratio of 14.9 ± 1.2 (7.02–100.59) on average. The soil hardness averaged 2.29 ± 0.09 MPa (0.66–4.92 MPa; Table S11). The soils within the nesting sites contained 9.65 ± 9.6 mg potassium (0.99–79.3 mg) and 16.9 ± 12.3 mg phosphorus (1.32–61 mg). Most nesting sites showed a high proportion of bare ground (mean 28.07% ± 1.17%/m^2^, 9.5%–65.5%/m^2^), with variable proportions of vegetation cover (mean 62.21% ± 23.79%/m^2^, 0%–99%/m^2^). The mean proportion of moss within nesting sites was 2.81% ± 0.45%/m^2^ (0%–16.89%/m^2^), and of litter 6.99% ± 0.41%/m^2^ (0%–18.88%/m^2^). Most nesting sites were located in (Gleyic) Cambisol or Brown Podzolic Soil, with only three nesting sites on Luvisol, occurring in the periphery of Braunschweig (Table S11). The soils of twelve study sites were anthropogenically modified, and especially the nesting sites located in parks were regularly found on hills from anthropogenic fillings.

**FIGURE 3:**
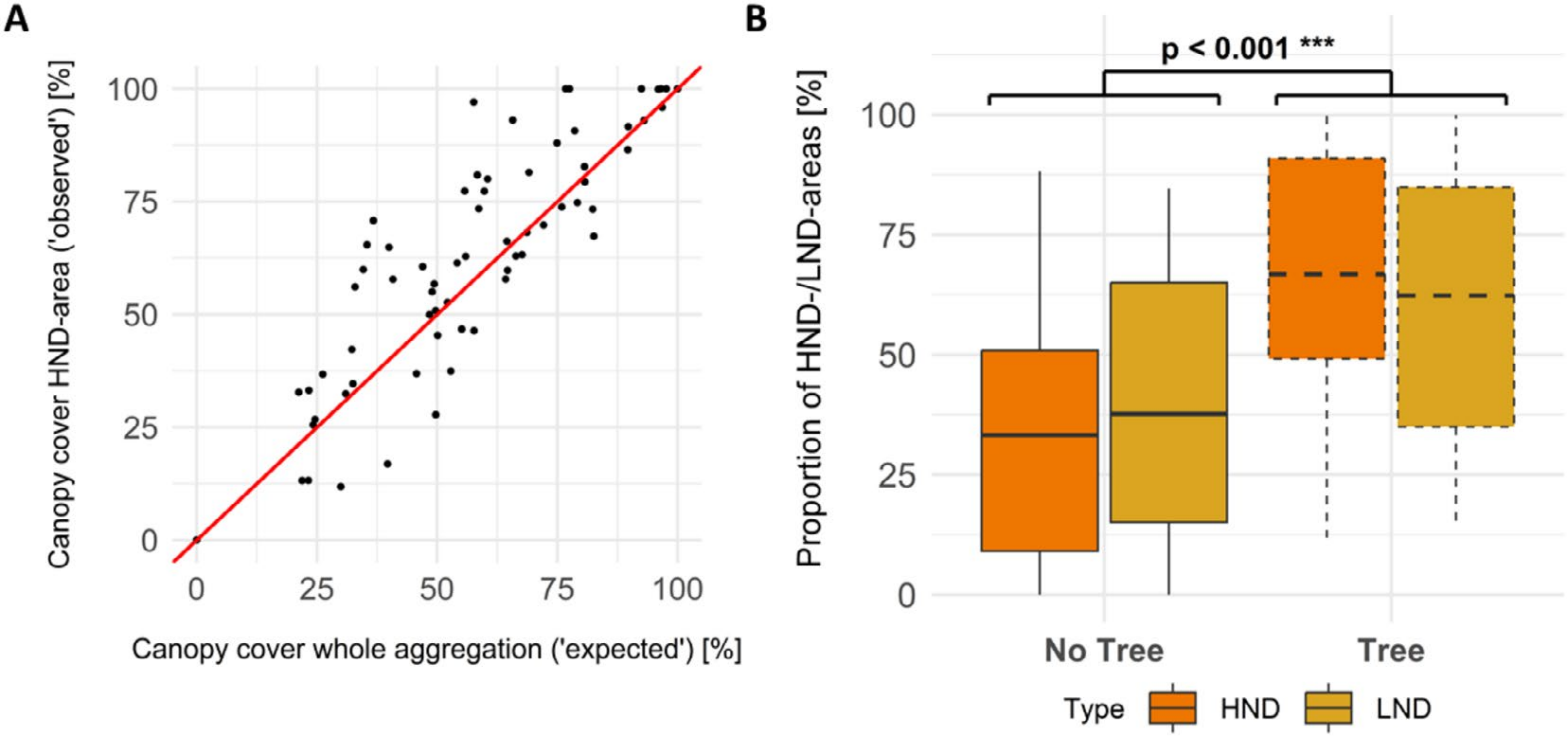
Canopy cover over HND‐areas. (A) More data points show a higher proportion of the HND‐area located under canopy cover (‘observed’; *n* = 41) compared to the proportion of the whole aggregation area (‘expected’; *n* = 24). The red line describes the case where both values match (*n* = 16). The corresponding paired Wilcoxon test showed that the observed values were higher than expected (*p* = 0.004). (B) Influence of the canopy cover (no tree = solid line, tree = dashed line) on the proportion of HND‐ and LND‐areas. A significantly higher proportion of both the HND‐ and LND‐areas was located under canopy cover (*p* < 0.001). Significance codes: *p* < 0.001 ***p < 0.01, **’*p* < 0.05, *p < 0.1.

Within HND‐areas, we found a higher proportion of bare ground (39.3% ± 23.9%; 1%–98%) compared to LND‐areas (16.9% ± 15.4%; 0.1%–98%), even in March before the bees' activity (HND: 33.7% ± 27.1%, 1%–98% vs. LND: 15.5% ± 17.8%, 0.1%–98%). However, the proportion of bare ground was lower and the difference less significant in March compared to April (Table S7; Figure S9). If a tree was present, an average of 67.13% ± 3.02% (11.83%–100%) of the HND‐area and of 62.49% ± 2.84% (21.25%–100%) of the whole aggregation area were located under canopy cover (Table S8). We did observe higher proportions of HND‐areas under trees than expected (*p* = 0.004 for the 1‐sided test; Figure 3A). The proportions of HND−/ and LND‐areas were significantly higher under canopy cover than in areas without tree cover (*p* < 0.001, *R* ^2^m = 0.7; Figure 3B). The interaction between plot type (HND vs. LND) and canopy cover (tree or no tree) was marginally significant (*p* = 0.055, *R* ^2^m = 0.7). However, the model results also indicate a trend that HND‐areas tended to have larger proportions under canopies compared to LND‐areas. We found 26 different tree species in or adjacent to *A. vaga* nesting sites, with a mean DBH of 0.43 ± 0.022 m (0.03–1.11 m; Table S9).

### 3.3 Comparison With Control

**FIGURE 4:**
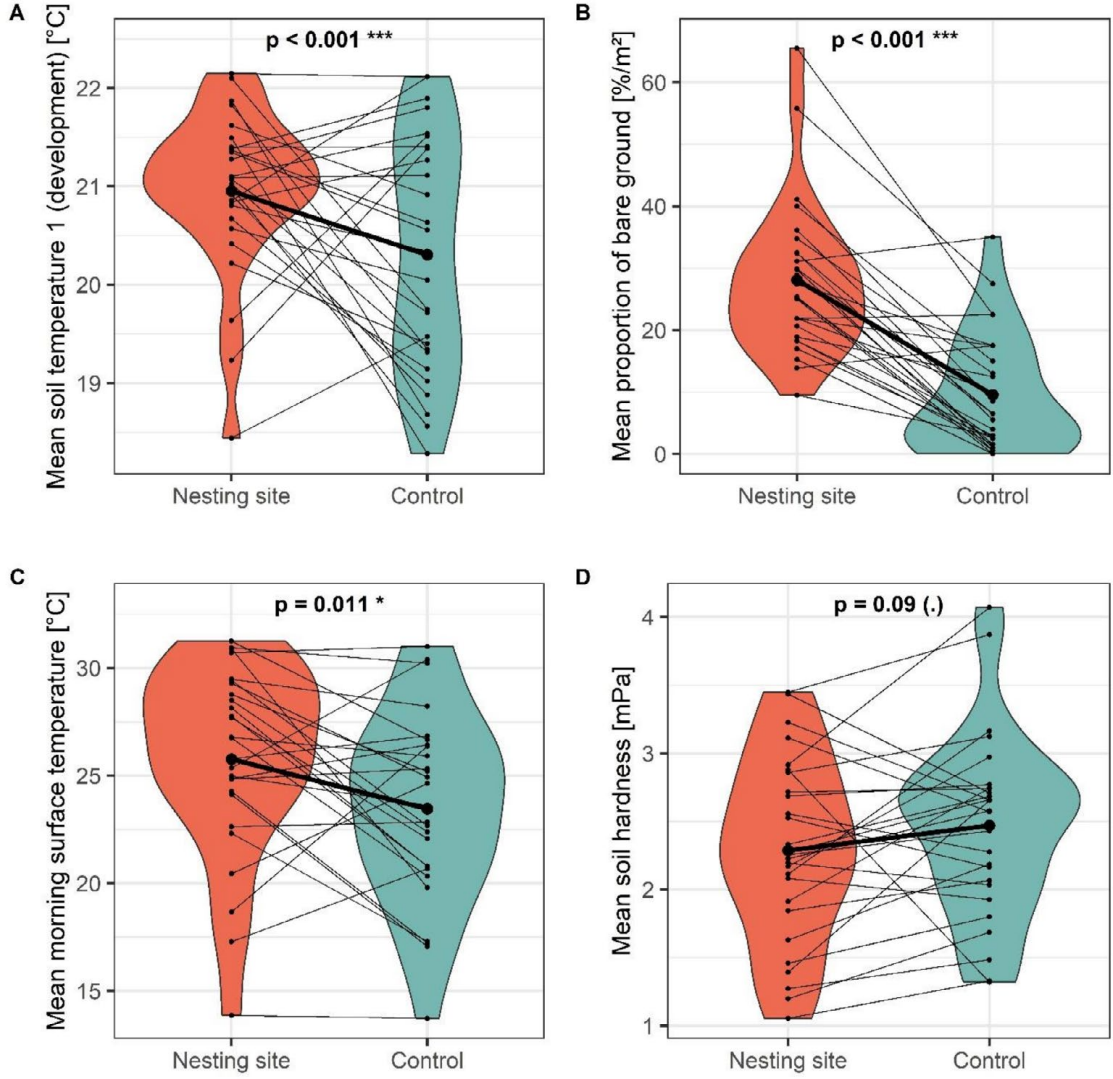
Violin plots for the comparison between nesting sites and controls: Influence of (A) the soil temperature during the development (°C), (B) the proportion of bare ground (%/m^2^), (C) the morning soil surface temperature (°C), and (D) the soil hardness (mPa). Dots show the mean values over all four depths per study site, bold lines represent mean values over all study sites. Lines connecting dots represent pairs of the nesting site and control plots per study site. Significance codes: *p* < 0.001, ***p < 0.01, **p < 0.05, *p < 0.1.

**FIGURE 5:**
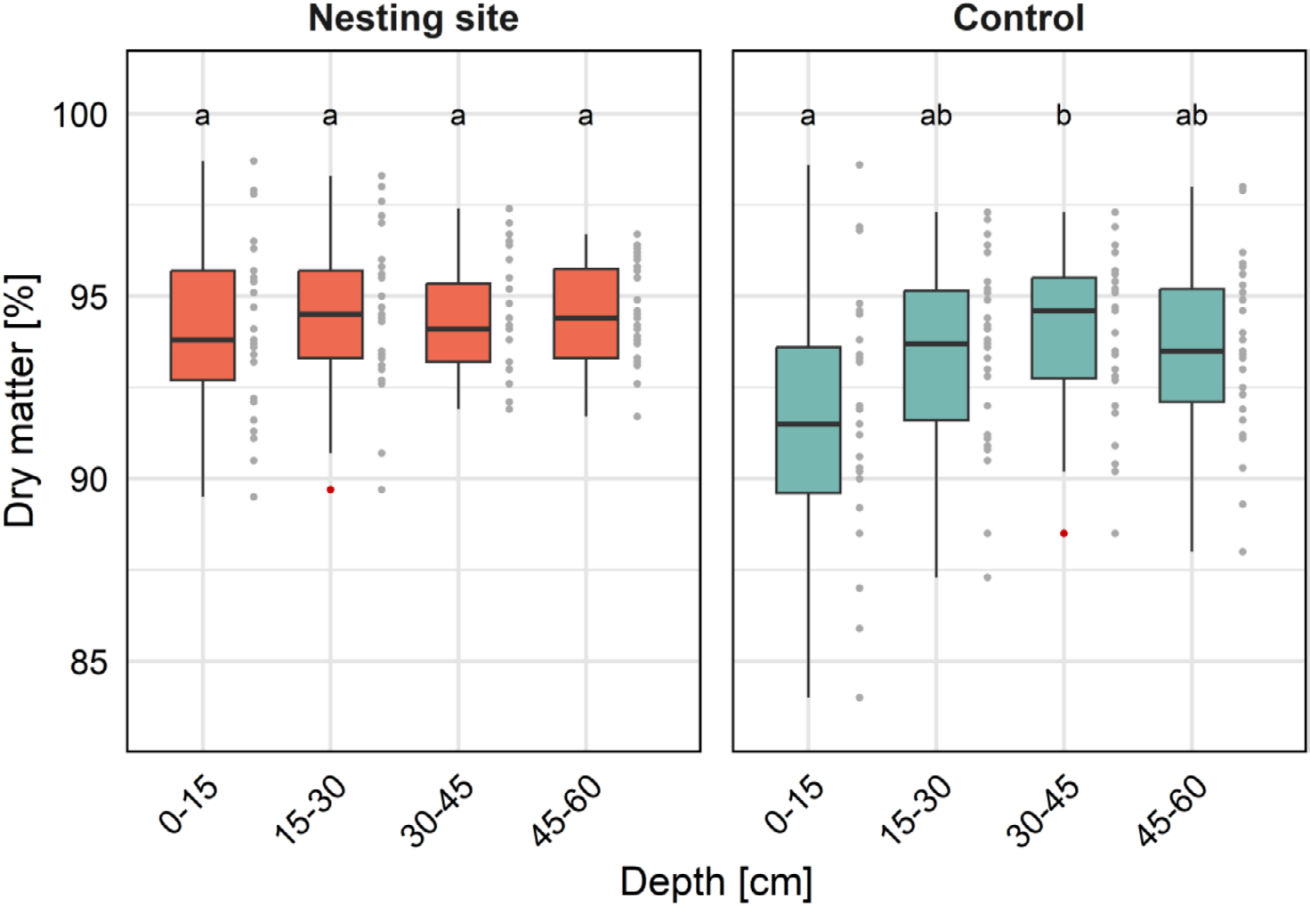
Interaction between the proportion of dry matter (%) and the soil depth (cm), for both the nesting sites and controls. Outliers are colored in red; grouping letters from the post hoc test per plot type are presented above. The proportion of dry matter of the nesting sites significantly differed from the controls within 0–15 cm. (*p* = 0.005; see also Figure S10).

The main factors differing between the nesting sites and controls were soil temperature during development (*p* < 0.001, *R* ^2^m = 0.23; Figure 4A), proportion of bare ground (*p* < 0.001, *R* ^2^m = 0.7; Figure 4B), morning soil surface temperature (*p* = 0.011, *R* ^2^m = 0.7; Figure 4C), and soil hardness (*p* = 0.09, *R* ^2^m = 0.23; Figure 4D). The interaction between the proportion of dry matter and soil depth was statistically significant: The soil in the controls contained more water (i.e., less dry matter), especially within the top 0–15 cm (*p* = 0.004; *R* ^2^m = 0.23; Figure 5 and Figure S10) than in plots within nesting sites, while no interaction with soil depth was found for any other parameter. There was no significant difference in the soil texture between nesting sites and controls.

### 3.4 Soil Parameters

**FIGURE 6:**
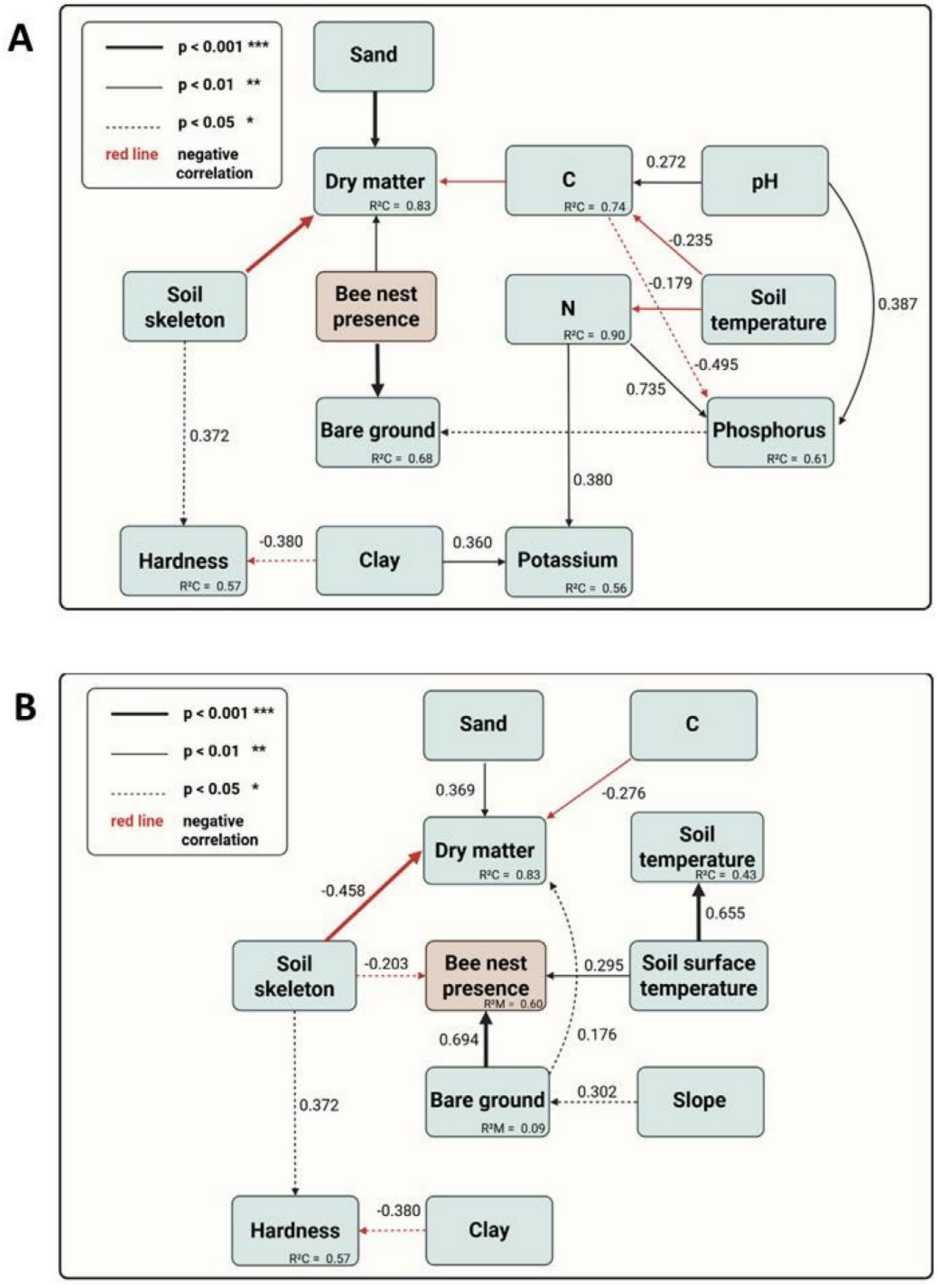
Directed acyclic graphs of the results from (A) SEM 1, with the presence of bee nests as **explanatory** variable, and (B) from SEM 2, with the nest presence as **dependent** variable. Only significant results are shown (*p* < 0.001 in bold lines, *p* < 0.01 in thin lines, *p* < 0.05 in dashed lines). Negative correlations are colored in red. The conditional *R* ^2^ values as well as the standardized estimates are shown (only calculated for paths where no binary variables were included).

The SEMs showed several significant relationships between the parameters (Tables S2, S3), illustrated in two Directed Acyclic Graphs (DAGs) in Figure 6A,B. Most correlations can be attributed to physical interactions, while some are driven by biological processes, i.e., the presence of bee nests. This first DAG (Figure 6A) shows the results of SEM 1 with the nest presence (plot type ‘nesting site’ vs. ‘control’) as an explanatory variable (Fisher's C = 98.149, *p* = 0.311). Here, the nest presence had a significant influence on the proportion of bare ground (*p* < 0.001) and dry matter (*p* < 0.001). The second DAG (Figure 6B) shows the results from SEM 2 with the nest presence as a dependent variable (Fisher's C = 58.69, *p* = 0.45). Here, the nest presence (plot type ‘nesting site’ vs. ‘control’) is explained by the proportion of bare ground (*p* < 0.001) and soil skeleton (*p* = 0.028), as well as the soil surface temperature (*p* = 0.002).

### 3.5 Experimental Bare Ground Plots

The artificially created bare ground plots were rarely colonized by *A. vaga* (Table S10). On twelve of the 39 plots, we found nests of *A. vaga* , but mainly in very small numbers between one and four (except for one plot with 12 nests). They were found in all three plot types (‘bare’, ‘sparse’, and even ‘control’). However, many nests of non‐target wild bee species were found on the plots, especially of small bee species. On three study sites, we found nest numbers between 100 and 140 nests per round and m^2^ (5/6 counts on ‘bare’ plots, one on ‘sparse’). Significantly more nests were found on the bare plots (*p* < 0.001; *R* ^2^c = 0.9; mean 32.3 ± 45.9 nests/m^2^) than on sparsely vegetated plots (mean 11.8 ± 29.6 nests/m^2^) or control plots (mean 6.36 ± 13.4 nests/m^2^; Figure S11A). The nest number of small bee species was negatively affected by vegetation cover (*p* < 0.001; *R* ^2^c = 0.88; Figure S11B). Specimens caught on the plots belonged to the following species: *Lasioglossum pauxillum* (*n* = 4), *Andrena minutula* (*n* = 1), and *Nomada flavoguttata* (*n* = 1). As not from all nest individuals could be sampled and determined, it is likely that more bee species nested on the experimental bare ground plots.

## 4 Discussion

In our study, we describe in great detail the nesting site characteristics of *A. vaga* as a model species for early emerging bees. Most interestingly, we detected a tendency of *A. vaga* to nest under canopy cover, a parameter presumably overlooked in previous nesting site descriptions. Only a few other authors have also reported, often rather casually or anecdotally, that certain bee or wasp species nest near or under trees, including *A. vaga* (Rezkova et al. 2012; Straka et al. 2014), *Andrena fulva* (Maher et al. 2019), or *Sphex ichneumoneus* (Brockmann 1979). In temperate regions, nesting under trees might be particularly beneficial in spring, as early in the season the absence of leaves allows sunlight to reach the nesting sites, while the canopy still provides shelter from rain and temperature extremes, such as late spring freezes (Fountain et al. 2023). These microclimatic conditions resemble those favorable for ephemeral spring flowers, which are also regularly found beneath trees (Figure S12; Tessier 2022).

Correspondingly, a warm soil (surface) temperature was one of the key factors indicating where *A. vaga* nests. This correlates with other authors’ descriptions of sun‐exposed nesting sites of *A. vaga* (Falk 1991; Bischoff 2003; Rezkova et al. 2012). Soil temperature affects bee life cycles and fitness in various ways (Antoine and Forrest 2021), influencing larval development (Forrest and Chisholm 2017), offspring survival (Ostap‐Chec et al. 2021), sex ratio (Soucy 2002), and adult body size (Soucy 2002; Kierat et al. 2017). Although *A. vaga* is able to moderately increase its body temperature through muscle activity (Rezkova et al. 2012), it is an ectothermic insect, requiring air temperatures above 10°C for flight (Westrich 2019). Warm soil surface temperatures therefore facilitate early foraging and rapid nest construction (Weissel et al. 2006). Nesting on slopes, as observed for *A. vaga* (Figure S19, Table S6), may represent a strategy to increase soil temperature (Antoine and Forrest 2021), although some nesting sites of *A. vaga* were also located in flat areas (Figure S20; see also Vleugel 1947; Ulrich 1956; Hallmen 1990; Bischoff 2003; Fellendorf et al. 2004; Rezkova et al. 2012). The mean morning soil surface temperature proved to be a better predictor of nesting site suitability than daily means. In accordance, Sakagami and Hayashida (1961) found nests of *Halictus duplex* preferably located in areas sun‐exposed during the early morning, even if the area became shaded and therefore less favorable later during the day. Most slopes colonized by *A. vaga* had a south‐eastern to south‐western exposure, thus receiving morning sun, while only a few exceptions faced west (cf. Vleugel 1947). Despite the preference of early spring bee species for warm, sun‐exposed nesting sites, nesting under single trees may likewise protect bees against heat stress in warmer periods or regions (Edmondson et al. 2016; Cheela et al. 2021).

In addition to microclimatic factors, bees might nest within the deep root network of trees (Figure S13 left) due to the reduced risk of tunnel collapse, which is particularly important at high nest densities. Although roots can damage brood cells (Wuellner 1999), the brood cell walls of *A. vaga* are comparatively well consolidated (see Video S1), which is why roots probably intrude into the brood cells only occasionally (Figure S13 right). As most trees on or near nesting sites were deciduous trees, leaf litter does not seem to bother *A. vaga* (Figure S14). Although nesting sites were often located near *Tilia cordata* and *Quercus robur*, no preference for specific tree species can be inferred due to the broad range of tree species documented at nesting sites (Table S9). Thus, it seems unlikely that bees benefit from microorganisms or root exudates associated with specific tree species. In cases where aggregations declined during our study, size reductions occurred in areas without canopy cover, suggesting that trees offer protection against negative impacts. For example, the trees' root systems might have prevented nest excavation by predators. Unfortunately, we do not know the aggregations' age, but we assume that canopy‐covered areas might serve as ‘founding areas’ for new nesting sites. Most nesting sites were located on the south‐facing side of trees, where they are sun‐exposed. Nevertheless, the largest aggregations extended to the northern side of trees, but at lower nest densities, suggesting that bees may colonize less suitable areas as nesting pressure increases.

The tendency to nest under trees can also be associated with a higher bare ground availability (Fountain et al. 2023), while still providing a solid soil structure, otherwise maintained by vegetation cover (Maher et al. 2019; Fountain et al. 2023). We identified bare ground as one of the key parameters determining where *A. vaga* nests (cf. Osgood Jr 1972), consistent with previous nesting site descriptions of *A. vaga* (Falk 1991; Bischoff 2003; Fellendorf et al. 2004; Straka et al. 2014), and many other ground‐nesting bee species (e.g., Potts et al. 2005; Lybrand et al. 2020; Antoine et al. 2025). Sparse or absent vegetation may be advantageous, as a dense root network and felted vegetation in the upper soil layer can otherwise obstruct nest excavation (Osgood Jr 1972; Wuellner 1999), and might decrease sun exposure. Consistent with Fountain et al. (2023), moss did not appear to deter *A. vaga* from nesting (Figure S15), possibly due to a shallow root system and growth.

However, there is ongoing debate whether ground‐nesting bees prefer soils with sparse vegetation cover or whether nests are simply more detectable on bare ground (Potts and Willmer 1997; Maher et al. 2019; Harmon‐Threatt 2020). In our first SEM, the presence of bee nests explained the availability of bare ground, suggesting a bidirectional relationship: As bees destroy roots during nest excavation and pile significant amounts of soil on small plants (Watanabe 1998; Cane 2003), they might suppress vegetation growth, especially in such dense nest aggregations as found for *A. vaga* . Since the soils *A. vaga* colonizes are mostly dry and sandy, vegetation recovery is slow (Osman 2018). This likely explains why we can still find higher proportions of bare ground in March, before the bees' activity. Thus, the assumption that bees generally prefer bare ground might be biased by observations of nest aggregations, easier to locate than single nests. Further experiments are needed to determine the direction of the relationship between bee nests and bare ground availability, as we were not able to explain the causality of our observations with the help of our SEMs or experimental bare ground plots.

Although bare ground availability was a strong predictor in our analysis, vegetation removal on experimental plots did not lead to colonization by *A. vaga* . Some of the study sites exhibited high nest densities, which would have made an expansion of the colonized area into the experimental bare ground plots expectable. Social aspects may have prevented individuals from nesting in the newly created, uncolonized plots, as bees are often attracted by the presence of conspecifics (Polidori et al. 2008; Batsleer et al. 2022). Additionally, although the plots were located near nesting site edges (2–5 m), this distance might still have been too long, as some emerging *A. vaga* females establish nests in close proximity to their natal nests (Malyshev 1926), often only a few centimeters apart—a phenomenon known as philopatrie (Yanega 1990). On the other hand, the plots might have been too close, as the marked bees in the mark‐recapture study of Bischoff et al. (2003) did not colonize new or established nesting sites at a distance of 100–200 m. Thus, bare ground availability does not fully explain nesting site selection of *A. vaga* , which must be considered within a complex interplay of abiotic and biotic factors.

Future studies should test whether establishing bare ground plots inside nesting sites attracts bees for nesting, since nest density increased with higher bare ground availability (Figure S9). Additionally, temporal factors may play a role in colonization. A long‐term study of these plots could provide new insights, as *A. vaga* may require time to establish nests in newly created structures. Comparable to our results, Fountain et al. (2023) primarily observed small *Lasioglossum* species nesting on newly created nesting plots, while most species, including large *Andrena* species, only nested on long‐established plots. On some plots, we found exceptionally high nest numbers of small ground‐nesting bees, indicating that conditions were generally suitable. Although it remains unclear whether the species nested in these areas previously, we found a strong preference for the plots with no or sparse vegetation cover. Even on sparsely vegetated plots, *Lasioglossum* nests were concentrated on bare ground patches, e.g., those created by moles, supporting previous findings that even small‐scale measures can promote ground‐nesting bees (cf. Fortel et al. 2016; Gardein et al. 2022). These findings further highlight the relevance of bare ground plots as a nesting resource for various wild bee species (Gregory and Wright 2005; Nichols et al. 2020).

Bare ground can facilitate the evaporation of the soil. We observed significantly higher proportions of dry matter and therefore less moisture in the top soil of nesting sites than in controls, comparable to the results of Pane and Harmon‐Threatt (2017) and Osgood Jr (1972). Moisture increases the risk of brood cell and provision spoilage (Fellendorf et al. 2004), which is why bees seem to avoid soils that are too moist, being able to detect humidity (Danforth et al. 2019). Although *A. vaga* colonizes floodplains, prolonged submergence of their nesting sites can lead to local extinctions, likely due to a lower oxygen availability (Ulrich 1956; Fellendorf et al. 2004). Following a high water event in Braunschweig in December 2023, we assessed the flooding risk of the studied nesting sites using maps of statutorily defined flood‐prone areas (Stadt Braunschweig 2022). Probably due to a high *Salix* tree abundance within flooding areas (Neyns et al. 2025), most nesting sites were located near water bodies and thus close to food resources, but remained outside the designated hazardous areas (Figure S16). Still, a balanced soil moisture is essential, as it softens the soil, facilitating brood cell excavation (Potts and Willmer 1997), and it supports larval weight gain (May 1972). In our first SEM, the presence of bee nests contributed to a higher proportion of dry matter. *Andrena vaga* buries more or less vertical tunnels, up to 60 cm deep and with a diameter of more than one centimeter (Westrich 2019), connecting the soil surface with the subsoil, and therefore likely increasing soil drainage (Tschanz et al. 2025).

Soil moisture is partly explained by texture due to a decreased water capacity of sandy soils (Osman 2018). The soils in Braunschweig contained high proportions of sand and were mainly quite homogeneous across depths (Fig. S17 left). However, they were less sandy and contained more clay compared to the ten *A. vaga* nesting sites Bischoff (2001) described in her study. Thus, nesting site descriptions from several regions are needed to cover the entire range a species tolerates. *Andrena vaga* nests in soils with lower sand and higher silt proportions compared to other bees, such as *Colletes cunicularius* (Bischoff 2001; A. Kratochwil (personal communication)). This highlights the fact that a description of nesting sites as ‘sandy’, as it is traditionally used to describe nesting sites of *A. vaga* , is too vague. A higher proportion of clay might be necessary for *A. vaga* given their wide tunnels and high nest densities (Brockmann 1979). Even though we found nesting sites with high clay proportions (study site 27, see Figure S17 right), *A. vaga* did not nest in (sandy) clay loam (Figure 2), although clay soils were available in the landscape of Braunschweig (BGR 2007). This observation is consistent with earlier reports (Vleugel 1947; but see Westrich 2019). Controls exhibited slightly more sand and less silt compared to the nesting sites (Figure S18), which might explain nesting site selection (Harmon‐Threatt 2020; Antoine and Forrest 2021), but this difference was not statistically significant. Even though heterogeneity in soil texture can occur on very small scales (Liu et al. 2018), the proximity of nesting sites and controls probably explained the missing difference in texture.

Soils within nesting sites exhibited lower penetration resistance than within controls, probably due to a lower soil skeleton content and higher clay proportions (SEM 1 & 2). Under moist conditions, clay can facilitate penetration, while it hardens when desiccated. The bees' digging activity likely contributed to the loose soil structure observed within the nesting sites (Tschanz et al. 2025), although there was no significant path in our SEM. One possible explanation is that penetration resistance was measured in March, before the bees' activity, and that the effect does not persist over longer periods. Therefore, repeated measurements, e.g., before, during, and after the bees' activity period, are recommended. It is unclear whether the soil in the nesting sites was also less consolidated. In accordance with other studies (Vleugel 1947; Exeler et al. 2008), at least two of our studied nesting sites were regularly driven over, and most parks and backyards are frequently trespassed. Even on extensive grasslands, the bees nested on and along paths. We therefore suspect *A. vaga* to tolerate these stressors, as some authors also mention that *A. vaga* nests in harder soils compared to other species (Bischoff 2000; Westrich 2019). Although compacted soils hinder nest excavation, they also inhibit excavation by predators (Antoine and Forrest 2021) and can prevent nest collapse (Potts and Willmer 1997).

The role of other soil characteristics such as pH and organic material in the bees' nesting site selection remains unclear (Harmon‐Threatt 2020), with most studies only reporting ranges recorded in nesting sites (Osgood Jr 1972; Potts and Willmer 1997; Polidori et al. 2010; Shebl 2017). However, Osgood Jr (1972) found less organic matter in the upper soil horizon of the examined nesting areas to be the most important factor differentiating them from control areas, presumably because high organic content impedes the penetration by bees. In our samples, topsoil horizons were quite narrow, roughly 5–10 cm deep, which is typical for sandy grasslands. Compared to the mean C:N ratio of 17 (20–30 cm depth) in *A. vaga* nesting sites analyzed by Bischoff (2001), we calculated a lower mean C:N ratio of 13.77 (15–30 cm depth). However, the ratio in this depth ranged from 9.54 to 26.45 among nesting sites, likewise indicating that *A. vaga* tolerates a broad range in some soil parameters.

Although nutrient levels did not correlate with bee nest presence in our first SEM, we found higher ratios of phosphorus and potassium in nesting sites compared to controls (see Gardein et al. 2025), particularly within 15–45 cm, where brood cells were primarily found. As willow pollen, but also nectar, contain high amounts of nutrients like potassium (Stanciu et al. 2011), unconsumed provisions might have enriched the soil with these nutrients. Additionally, exocrine gland secretions used for brood cell linings (Cane 1981), dead bees, and a sparse vegetation cover can contribute to nutrient accumulation. However, it cannot be excluded that the elevated values may have resulted from fertilization of the sites.

The environmental conditions in Braunschweig seem to match the biological requirements of *A. vaga* , likely explaining why it is common here (Weber et al. 2023). Besides suitable soil conditions, *Salix* trees are very abundant in Braunschweig and occur, for most nesting sites, in sufficient numbers within a 300 m radius (Neyns et al. 2025). With a mean of 265.04 m^2^ and a maximum of 978 m^2^, *A. vaga* populations in Braunschweig covered larger areas than previously reported (67 m^2^ Ulrich 1956; 160–240 m^2^ Fellendorf et al. 2004). Additionally, the highest maximum nest number of 280 nests per m^2^ recorded in our study exceeded prior references of ‘more than 30’ (Westrich 2019) or ‘up to 50 females per m^2^’ (Hallmen 1990). Most nesting sites and HND‐areas showed stable locations over the three study years, indicating that the bees were not exposed to major stressors that would have provoked a relocation, e.g., parasite and pathogen accumulation, an increased soil instability (Antonini et al. 2003; Harmon‐Threatt 2020), or misguided grassland management (Hallmen 1990). However, some aggregations showed (drastic) declines, potentially explained by increased mole activity, the use of a robot lawn mower, ceased mowing, or the more intensive use of the green space for city festivals or market stalls. Most aggregation sizes fluctuated over the three years, probably due to parasite–host interactions (Volterra 1928) or a biennial pollen production cycle of willows (Weryszko‐Chmielewska et al. 2017).

Our study shows that urban landscapes can provide suitable bee habitats where even highly specialized bees like *A. vaga* can establish large populations (cf. Banaszak‐Cibicka and Żmihorski 2012). Additionally, we can confirm that green urban infrastructure such as cemeteries, parks, and lawns can provide high‐quality nesting structures (Baldock et al. 2019; Weber et al. 2023). During the last century, the natural habitat of *A. vaga* has been degraded by river canalization, wetland drainage, and dry grassland cultivation (Exeler et al. 2008), resulting in decreasing floral host (*Salix* spp.) and nesting site availability. As all nesting sites of our study were either regularly mown and/or trespassed, moderate anthropogenic disturbances within urban environments might have replaced natural dynamic processes like flooding or erosion that otherwise create nesting sites (Winfree et al. 2007; Prendergast et al. 2022). In contrast to natural nesting sites that are often only temporary due to succession, continuous human‐induced disturbances may ensure a sustained availability of suitable nesting sites.

## 5 Conclusion

Despite being a relatively well‐described species, we provide new insights into *A. vaga'*s nesting biology, including a preference for nesting under canopy cover—an overlooked aspect that should be considered when studying other ground‐nesting bees. In our study, we use standardized soil science methods that we recommend for enabling comparisons within and across bee species. Our results show that bee species can tolerate a range of nesting site conditions, highlighting the importance of studying multiple nesting sites across environmental contexts and regions. Despite the considerable difficulty in spatially defining the control, comparisons between colonized and uncolonized areas, a rarely applied approach, provide valuable information on habitat preferences and potential impacts of bee activity. Nevertheless, our study did not resolve a “chicken‐and‐egg dilemma”: Do the bees prefer to nest in areas with sparse vegetation and high drainage, or is their activity the reason for these characteristics? Future studies should monitor the colonization of new nesting grounds and track changes in soil properties to understand the bees' role as ecosystem engineers.

Bare ground was indicated as a key parameter for nesting sites of *A. vaga* and other ground‐nesting bees, but it is often undesired and considered anaesthetic. The removal of plant material and the surrender of fertilizer or (bark) mulch application could be a first important step to maintain bare ground in public spaces. The trespassing of public green spaces should not be prohibited, accepting small‐scale bare ground patches and paths to arise. Frequent mowing of selected subareas in public green spaces outside of bees' peak flight times, e.g., in the evening, can increase bare ground availability and enhance sun exposure to the soil. Ideally, this should be carried out under single, exposed trees or along the south‐facing edges of tree groupings. Other parts of green spaces should be less frequently mown to allow plants to flower, providing nearby floral (nectar) resources.

## Author Contributions

**Hanna Gardein:** conceptualization (lead), data curation (lead), formal analysis (lead), investigation (lead), methodology (lead), project administration (lead), validation (lead), visualization (lead), writing – original draft (lead). **Tim Diekötter:** conceptualization (supporting), methodology (supporting), supervision (supporting), validation (supporting), writing – review and editing (equal). **Elke Bloem:** data curation (supporting), investigation (equal), methodology (equal), resources (equal), validation (supporting), writing – review and editing (equal). **Henri Greil:** conceptualization (equal), funding acquisition (lead), methodology (equal), resources (equal), supervision (lead), validation (supporting), writing – review and editing (equal).

## Conflicts of Interest

The authors declare no conflicts of interest.

## Supporting information


**Video S1:** ece372327‐sup‐0001‐Video.mp4.

**Appendix S1:** ece372327‐sup‐0002‐AppendixS1.pdf.

## Data Availability

The soil sample data that support the findings of this study are openly available in OpenAgrar at https://doi.org/10.5073/20250525‐104821‐0. Additional data that support the findings of this study are available in the Supporting Information of this article.
