# Supplementary material for: Barefoot Trees for Bees: Nesting Site Characteristics of the Ground‐Nesting Bee Andrena vaga in an Urban Environment
Source: Ecol Evol. 2025 Oct 18;15(10):e72327. doi: 10.1002/ece3.72327 (PMC12535216; doi:10.1002/ece3.72327)
Supplement: Supplementary file 2 — Appendix S1: ece372327‐sup‐0002‐AppendixS1.pdf. [file ECE3-15-e72327-s001.pdf]

Supplement

Barefoot trees for bees: Nesting site characteristics of the ground-nesting bee *Andrena vaga* in an urban environment

Hanna Gardein, Tim Diekötter, Elke Bloem, Henri Greil

Full method soil samples

Soil samples were air-dried at room temperature and sieved through a 2 mm sieve. The proportion of the soil skeleton was determined by weighing out the fraction > 2 mm and the sieved fraction. Soil texture was determined partly according to DIN ISO 11277 but by using Atterberg cylinders for sedimentation and determination of the clay content. Shortly, 30 g of air-dried, 2 mm sieved soil was mixed with 100 mL deionized water. Afterwards, hydrogen peroxide (30% H_2O_2) was added in 5 mL proportions to destroy the organic matter and were left overnight to react. As long as the samples still foamed more H_2O_2 was added the next day. After 3-5 days of reaction time when no more reaction was observed samples were heated on a sand bath, the H_2O_2 was destroyed and the sample was concentrated to 100 mL. 20 mL of concentrated hydrochloric acid (32% HCl) was added to destroy lime and sesquioxides and were left for 1h to react. Afterwards, samples were centrifuged at 8000 U for 20 minutes and the clear supernatant was discarded. The pellet was washed two times with deionized water, centrifuged at 8000 U for 20 minutes, the clear supernatant was discarded and after two cleaning cycles the pellet was dried at 105 °C. From the dry pellet exactly 25 g was weight into a PE bottles, about 200 mL of deionized water and 25 mL of sodium metaphosphate (4% NaPO3) were added and samples were shaken overhead for 2 hours to disperse particles. Afterwards, samples were washed over a sieve tower consisting of four sieves, namely 0.630 µm, 0.200 µm, 0.063 µm and 0.020 µm. The fraction on the 0.630 µm sieve correspond to coarse sand, on the 0.200 µm sieve to medium sand and on the 0.063 µm sieve to fine sand. The fraction on the 0.020 µm sieve correspond to coarse silt. The washing solution containing medium and fine silt and clay was transferred into an Atterberg cylinder with a fill level of exactly 30 cm. Separation of the silt fraction (medium + fine silt) from the clay fraction was performed by sedimentation velocity. Silt particles sediment during 24 h while clay remain in solution. After 24 h the supernatant was discarded and the cylinder was filled up again to 30 cm with deionized water, shaken and again let sediment for 24 hours. This procedure was repeated until the supernatant was clear after 24 hours what indicated that no more clay was contained in the silt fraction. The silt fraction was dried at 105 °C and weight and the clay fraction was calculated as difference from 25 g of sample minus the sand and the silt fractions.

Soil pH and conductivity were determined in the same extract. Therefore, 10 g of air-dried and 2mm sieved soil were mixed and shake up by hand for 1 minute with 25 mL deionized water and were left then for 30 minutes to reach equilibrium. Samples were shaken again and left for 10 minutes to sediment. Afterwards, pH were measured by a Seven Multi pH meter (Mettler Toledo). For the determination of conductivity again 25 mL deionized water were added and samples were shaken on a horizontal shaker for 30 minutes. Afterwards samples were left for 15 minutes to sediment and conductivity was measured at 21.7 °C by a Seven Multi conductivity meter (Mettler Toledo). Soil dry matter content was determined by drying 10 g of fresh soil at 105 °C for 24 hours and by determine the difference in weight. The proportion of the dry matter and therefore the water content (in %) was calculated. From this dried soil total carbon (C) and nitrogen (N) were measured via dry combustion in a C:N analyzer (Vario Max Cube, Elementar, Langenselbold, Germany). Available soil potassium (K_2O) and phosphorus (P_2O_5) were determined in a calcium acetat lactat (CAL) extract according to Schüller (1969) and were analysed by Inductively Coupled Plasma - Optical Emission Spectrometry (ThermoFisher Scientific iCAP 6000 series, Cambridge, UK).

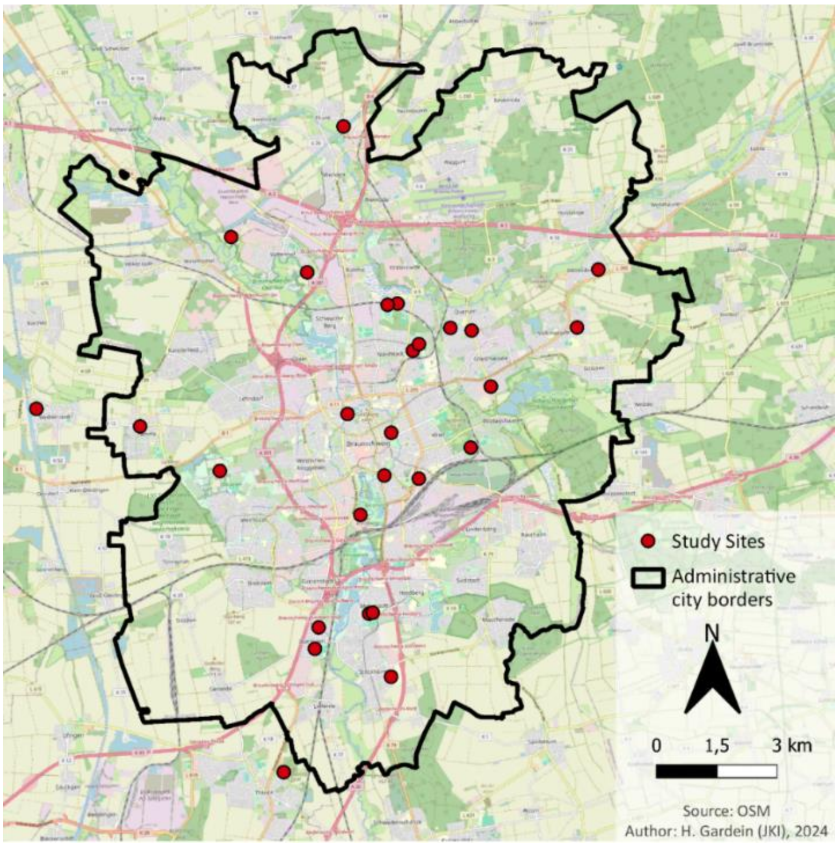

Figure S 1: Map of the 27 study sites within Braunschweig (red dots).

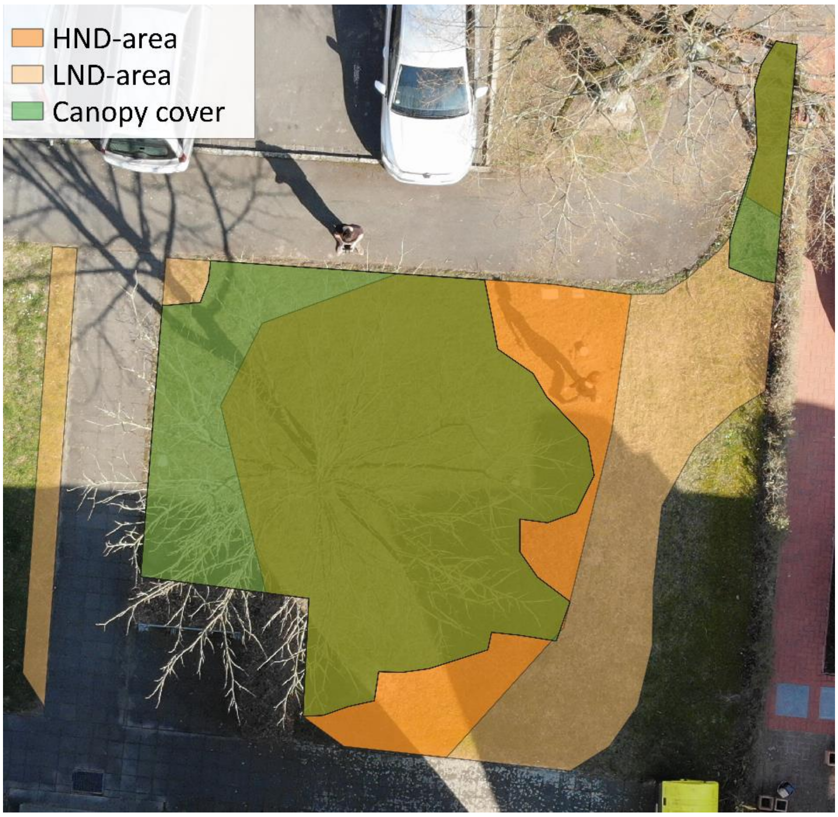

Figure S 2: Drone picture of study site 9 with estimated canopy cover (green) over the total aggregation (light orange) and HND-area (dark orange)

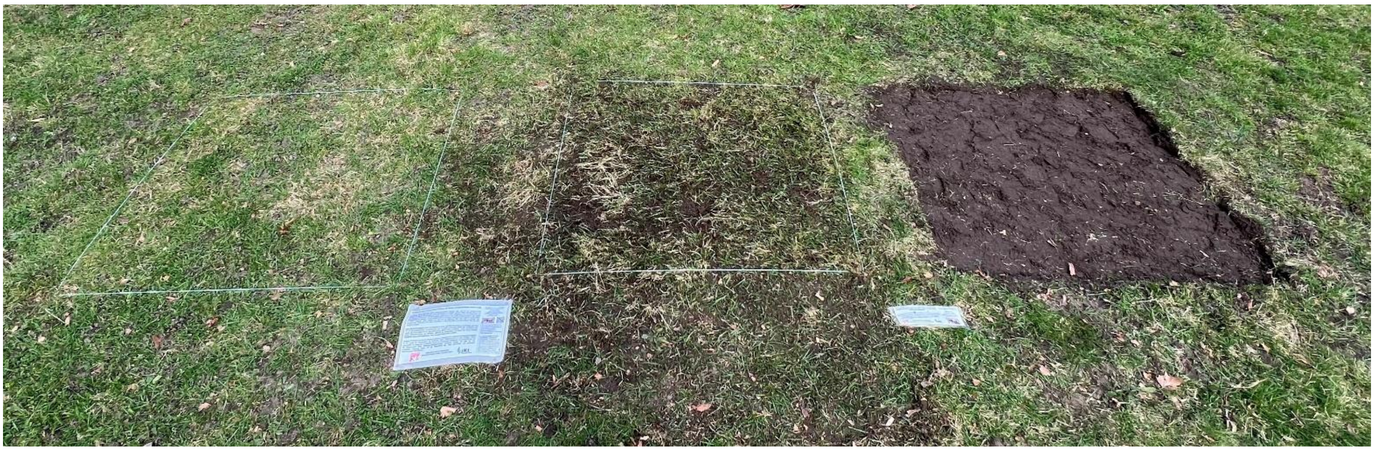

Figure S 3: Experimental setup of the artificial bare ground plots (study site 16). Left: 'control', middle: 'sparse', right: 'bare'

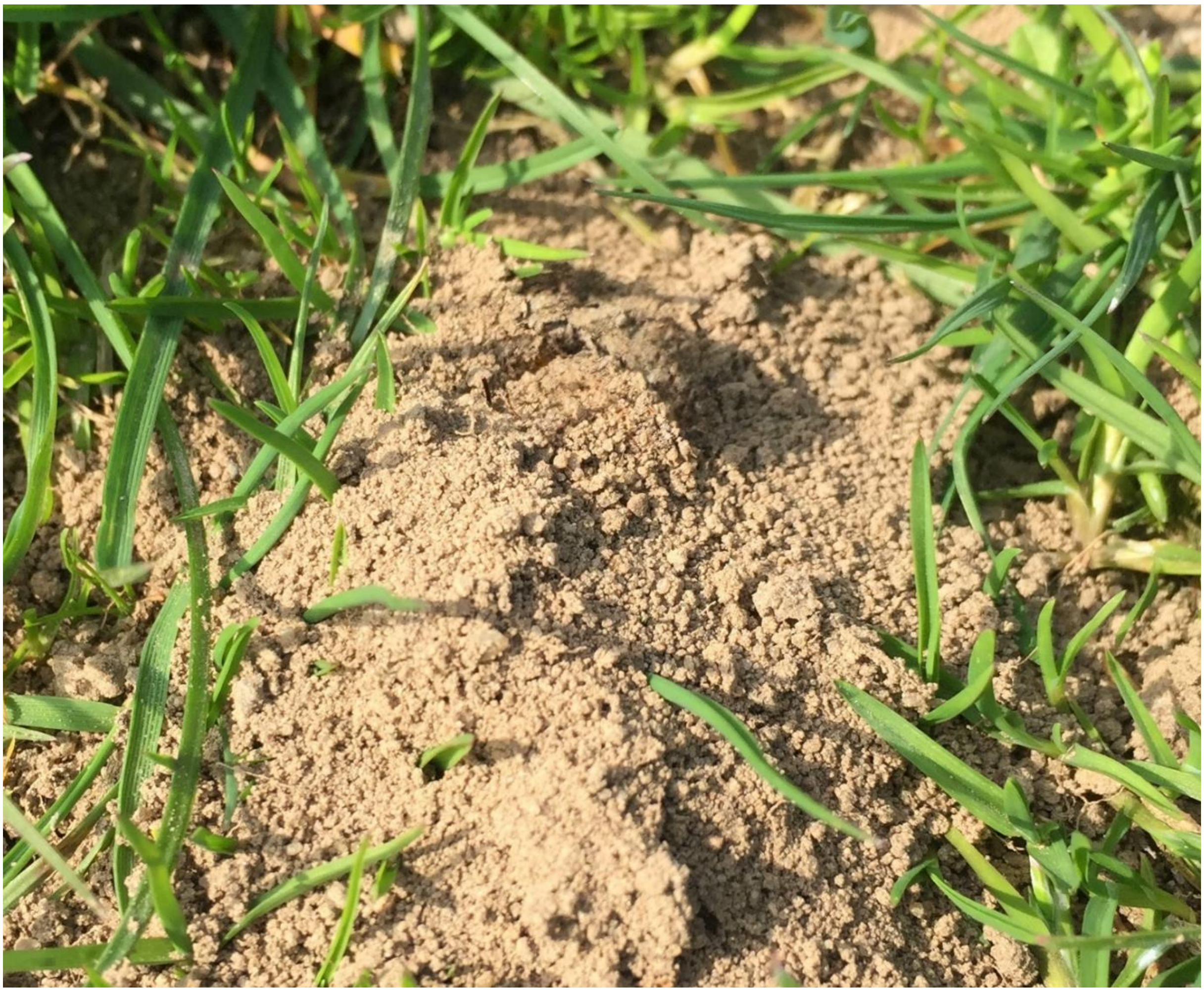

Figure S 4: Closed nest entrance of A. vaga

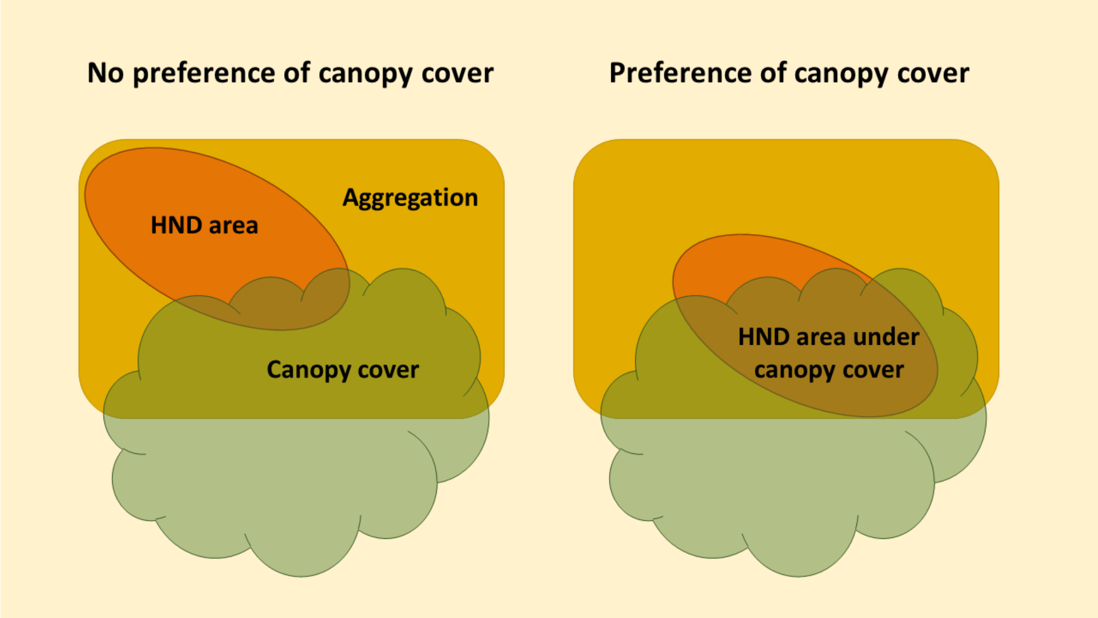

Figure S 5: Visualization of the question, whether the HND-areas were preferably located under canopy cover

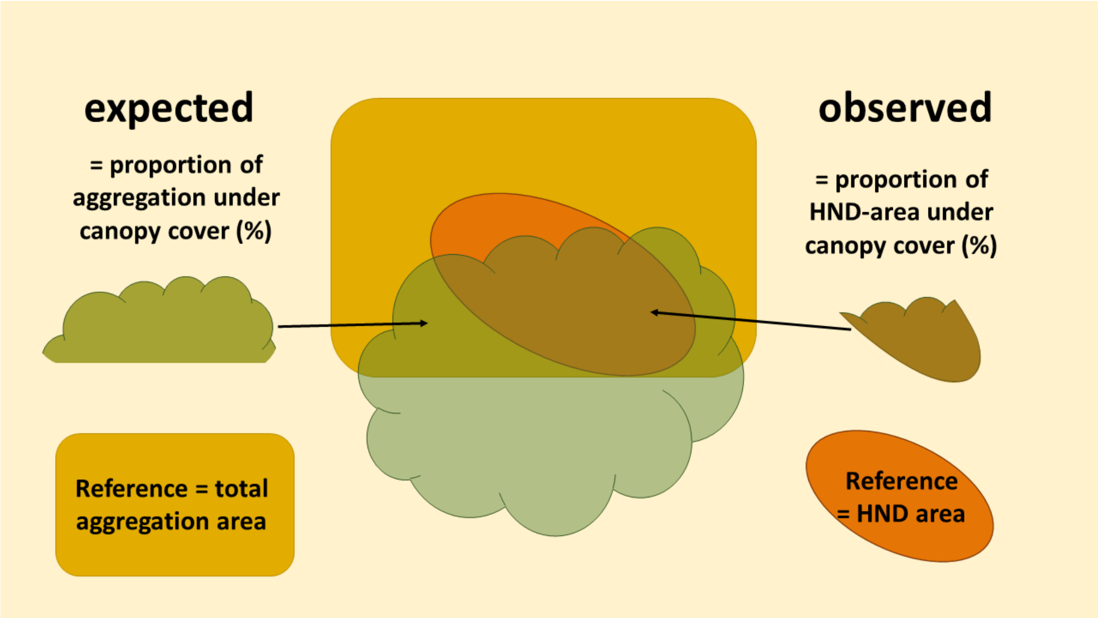

Figure S 6: Visualization of the expected vs. observed values: The proportion of the total aggregation area under canopy cover is determined as the expected value. It is compared to the observed proportion of the HND-area that is located under canopy cover.

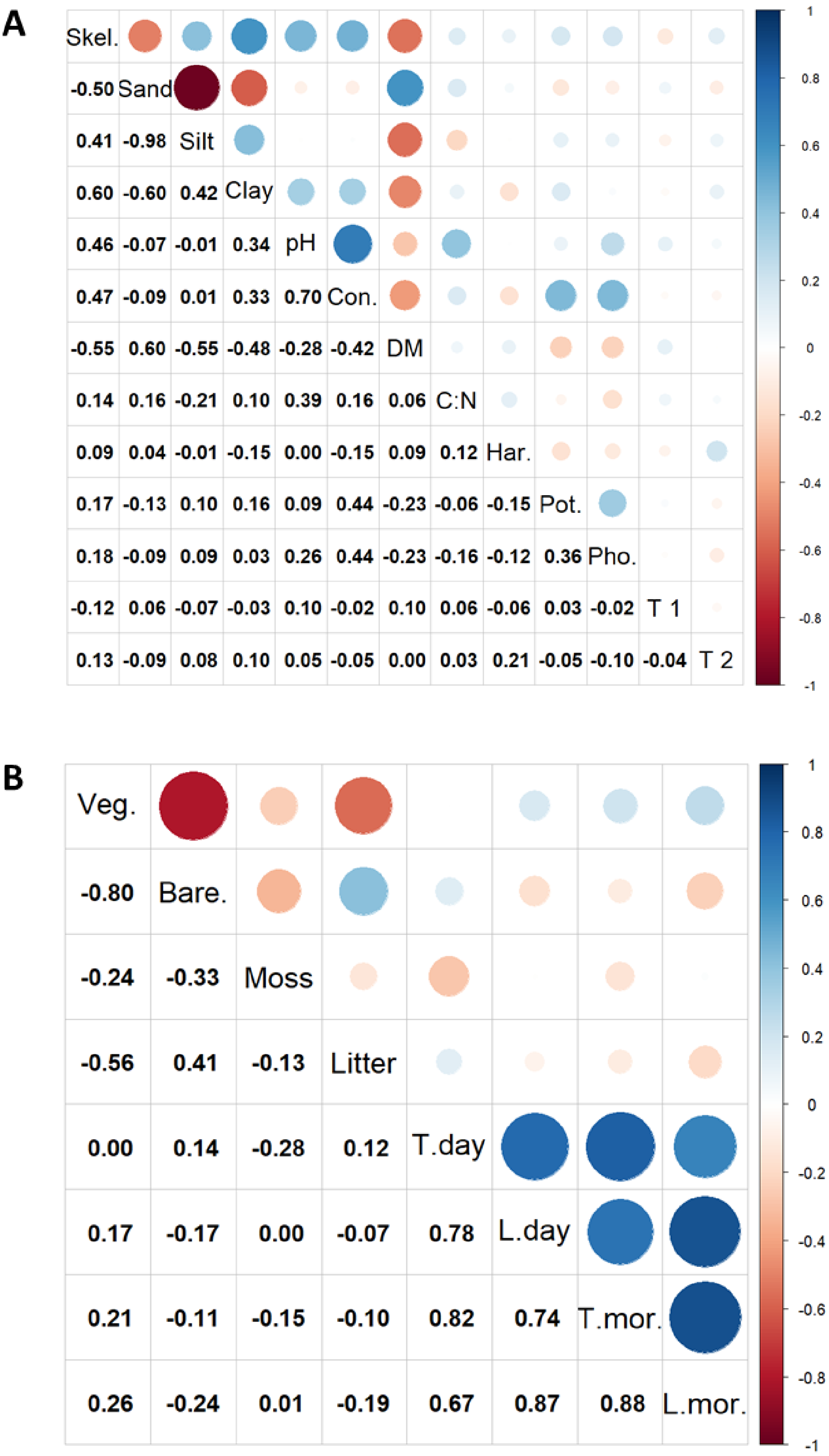

Figure S 7: Correlation matrices for the comparisons of A) the soil conditions (model m5) and B) the soil surface characteristics (model m6) between nesting sites and controls. Abbreviations refer to: Skel = Soil skeleton, Con. = conductivity, DM = dry matter, Har. = Hardness, Pot. = Potassium, Pho. = Phosphorus, T 1 = soil temperature 1, T2 = soil temperature 2, Veg. = vegetation, Bare. = bare ground, T. day = mean day soil surface temperature, L. day = mean day light intensity, T. mor = mean morning soil surface temperature, L. mor = mean morning light intensity

Table S 1: Overview of the statistical models. As many parameters correlated with another parameter (pH/conductivity, sand/silt, sand/clay, sand/dry matter, clay/soil skeleton, bareground/vegetation cover, and all soil surface temperatures and light intensities; see Fig S7), several models with possible combinations of variables were calculated and the best model was chosen using AIC, R^2 and residual fit.

Variable		Model	AIC
Vegetation April 22/23	Full model	M1.1 = glmmTMB (Plot.Type ~ Bareground + Litter + Moss + (1|Site.ID) + (1|Year), family = binomial, data = vegetation)	243.22
	Best model	M1 = glmmTMB (Plot.Type ~ Bareground + Litter, family = binomial, data = vegetation)	237.24
Vegetation March 23	Full model	M2.1 = glmmTMB (Plot.Type ~ Bareground + Moss + Litter + (1|Site.ID), family = binomial, data = vegetation2)	134.21
	Best model	M2 = glmmTMB (Plot.Type ~ Bareground + Litter, family = binomial, data = vegetation2)	130.75
Canopy 1	2-sided	M3a = wilcox.test (Tree$Observed, Tree$Expected, paired = TRUE)	
	1-sided	M3b = wilcox.test (Tree$Observed, Tree$Expected, paired = TRUE, alternative="greater")	
Canopy 2	Full model	M4.1 = glmmTMB (Area ~ Type*Tree + (1|Site.ID) + (1|Year), family = tweedie(link = "log"), data = Tree)	185.66
	Best model	M4 = glmmTMB (Area ~ Type*Tree , family = tweedie(link = "log"), data = Tree)	181.66
Aggregation vs. control 1	Full model 1	M5.1 = glmmTMB (Plot.Code ~ Depth*C:N + Depth*Dry matter + Depth*Soil skeleton + Depth*Hardness + Depth*Soil_temp_1 + Depth*Soil_temp_2 + (1|Site.ID), family = binomial, data = Soil)	302.48
	Full model 2	M5.2 = glmmTMB (Plot.Code ~ Depth*C:N + Depth*Dry matter + Depth*Clay + Depth*Hardness + Depth*Soil_temp_1 + Depth*Soil_temp_2 + (1|Site.ID), family = binomial, data = Soil)	309.81
	Full model 3	M5.3 = glmmTMB (Plot.Code ~ Depth*C:N + Depth*Sand + Depth*Soil skeleton + Depth*Hardness + Depth*Soil_temp_1 + Depth*Soil_temp_2 + (1|Site.ID), family = binomial, data = Soil)	313.96
	Full model 4	M5.4 = glmmTMB (Plot.Code ~ Depth*C:N + Depth*Sand + Depth*Clay + Depth*Hardness + Depth*Soil_temp_1 + Depth*Soil_temp_2 + (1|Site.ID), family = binomial, data = Soil)	326.50
	Best model	M5 = glmmTMB (Plot.Code ~ Depth:Dry matter + Hardness + Soil_temp_1, family = binomial, data = Soil)	277.70

Variable		Model	AIC
Aggregation vs. control 2	Full model 1	M6.1 = glmmTMB (Plot.Code ~ Bareground + Moss + Litter + Day_temp + (1|Site.ID), family = binomial, data = Soil)	53.99
	Full model 2	M6.2 = glmmTMB (Plot.Code ~ Bareground + Moss + Litter + Morning_temp + (1|Site.ID), family = binomial, data = Soil)	47.15
	Full model 3	M6.3 = glmmTMB (Plot.Code ~ Bareground + Moss + Litter + Day_light + (1|Site.ID), family = binomial, data = Soil)	54.91
	Full model 4	M6.4 = glmmTMB (Plot.Code ~ Bareground + Moss + Litter + Morning_light + (1|Site.ID), family = binomial, data = Soil)	49.50
	Best model	M6 = glmmTMB (Plot.Code ~ Bareground + Morning_temp, family = binomial, data = Soil)	40.02
Experimental plots 1	Full model	M7 = glmmTMB (Nest.number.small ~ Plot.type + (1|Site.ID) + (1|Round), family = tweedie(link = "log"), data = Nests)	465.58
	Best model	M7 = glmmTMB (Nest.number.small ~ Plot.type + (1|Site.ID), family = tweedie(link = "log"), data = Nests)	463.58
Experimental plots 2	Full model 1	M8.1 = glmmTMB (Nest.number.small ~ Bareground + sqrt(Moss) + sqrt(Litter) + (1|Site.ID) + (1|Round), family = tweedie(link = "log"), data = Nests)	464.78
	Best model	M8 = glmmTMB (Nest.number.small ~ Bareground + sqrt(Litter) + (1|Site.ID), family = tweedie(link = "log"), data = Nests)	462.78

SEM 1 - Full model

```
psem_model <- psem(
Sand %~~% Clay,
Clay %~~%  Skeleton,
C %~~% N,
Potassium %~~% Phosphate,
m.Bare = lmer(sqrt(Bareground) ~ Slope + Phosphate + Plot.Code + (1|Site.ID),
  data = Soil_mean),
m.DM = lmer(DryMatter ~ sqrt(Bareground) + Slope + Sand + C+ Skeleton + Surface_temp +
  Plot.Code + (1|Site.ID), data = Soil_mean),
m.Hardness = lmer (Hardness ~ DryMatter + Skeleton + Clay + Plot.Code + (1|Site.ID),
  data = Soil_mean),
m.N = lmer(N ~ pH + Soil_temp + Plot.Code + (1|Site.ID), data = Soil_mean),
m.C = lmer(C ~ pH +Soil_temp + Plot.Code + (1|Site.ID), data = Soil_mean),
m.Pot = lmer (log(Potassium) ~ C + N + Clay + Soil_temp + Plot.Code + (1|Site.ID), data = Soil_mean),
m.Phos = lmer(Phosphate ~ N + pH + C+ Soil_temp + Plot.Code + (1|Site.ID), data = Soil_mean))
```

SEM 1 - Best model

```
psem_model <- psem(
Sand %~~% Ton,
Ton %~~%  Skeleton_prozent,
C %~~% N,
Potassium %~~% Phosphate,
m.Bare = lmer(sqrt(Bareground) ~ Phosphate + Plot.Code + (1|Site.ID), data = Soil_mean),
m.DM = lmer(DryMatter ~ Sand + C+ Skeleton + Plot.Code + (1|Site.ID), data = Soil_mean),
m.Hardness = lmer (Hardness ~ Skeleton_prozent + Clay + Plot.Code + (1|Site.ID),
  data = Soil_mean),
m.N = lmer(N ~ Soil_temp + (1|Site.ID), data = Soil_mean),
m.C = lmer(C ~ pH +Soil_temp + (1|Site.ID), data = Soil_mean),
m.Pot = lmer (log(Potassium) ~ N + Clay + (1|Site.ID), data = Soil_mean),
m.Phos = lmer(Phosphate ~ N + pH + C+ (1|Site.ID), data = Soil_mean))
```

Table S 2: Statistical results of SEM 1

**Response**	**Predictor**	**Std.Error**	**P.Value**	**Std.Estimate**	
~~Sand	~~Clay	-	0	-0.5606	***
~~Clay	~~Skeleton	-	0	0.6447	***
~~C	~~N	-	0	0.5924	***
~~Potassium	~~Phosphate	-	0,0038	0.3623	**
sqrt(Bareground)	Phosphate	0.0195	0,021	-	*
sqrt(Bareground)	Plot.Code	-	0	-	***
sqrt(Bareground)	Plot.Code = 0	0.252	0	-	***
sqrt(Bareground)	Plot.Code = 1	0.252	0	-	***
DryMatter	Sand	0.025	5,00E-04	-	***
DryMatter	C	0.5092	0,0069	-	**
DryMatter	Skeleton	0.0225	4,00E-04	-	***
DryMatter	Plot.Code	-	0,0072	-	**
DryMatter	Plot.Code = 0	0.233	0	-	***
DryMatter	Plot.Code = 1	0.233	0	-	***
Hardness	Skeleton	0.0118	0,0202	0.3723	*
Hardness	Clay	0.06	0,0272	-0.38	*
N	Soil_temp	0.0012	0,005	-0.1789	**
C	pH	0.0601	0,0229	0.2721	*
C	Soil_temp	0.0306	0,0128	-0.2354	*
log(Potassium)	N	31.473	0,0073	0.3801	**
log(Potassium)	Clay	0.0365	0,0089	0.3599	**
Phosphate	N	906.028	0,0014	0.7348	**
Phosphate	pH	18.933	0,0086	0.3874	**
Phosphate	C	54.238	0,0229	-0.4946	*

SEM 2 - Full model

```
psem_model2 <- psem(
Clay %~~% Sand,
Clay %~~% Skeleton,
C %~~% N,
m.Bare = lmer (sqrt(Bareground) ~ Slope + Phosphate+ C + N + (1|Site.ID), data = Soil_mean),
m.DM = lmer (DryMatter ~ Slope + Sand + C + Skeleton + Surface_temp + sqrt(Bareground) +
  (1|Site.ID), data = Soil_mean),
m.Hardness = lmer (Hardness ~ DryMatter + Skeleton + Clay + (1|Site.ID), data = Soil_mean),
m.Temp = lmer (Soil_temp ~ Surface_temp + sqrt(Bareground) + DryMatter + C + Skeleton +
  (1|Site.ID), data = Soil_mean),
m.Temp2 = lmer (Surface_temp ~ Slope + sqrt(Bareground) + C + Skeleton + (1|Site.ID),
  data = Soil_mean),
m.Plot=lmer(Plot.Code ~ sqrt(Bareground)+ DryMatter + Hardness + Soil_temp +Surface_temp +
  (1|Site.ID), data=Soil_mean))
```

SEM 2 - Best model

```
psem_model2 <- psem(
Clay %~~% Sand,
Clay %~~% Skeleton,
C %~~% N,
m.Bare = lm (sqrt(Bareground) ~ Slope, data = Soil_mean),
m.DM = lmer (DryMatter ~ Sand + C + Skeleton + sqrt(Bareground) + (1|Site.ID),
  data = Soil_mean),
m.Hardness = lmer (Hardness ~ Skeleton + Clay + (1|Site.ID), data = Soil_mean),
m.Temp = lmer (Soil_temp ~ Surface_temp + (1|Site.ID), data = Soil_mean),
m.Plot=lm(Plot.Code ~ sqrt(Bareground)+ Skeleton + Surface_temp, data=Soil_mean))
```

Table S 3: Statistical results of SEM 2

Response	Predictor	Std.Error	P.Value	Std.Estimate	
~~Clay	~~Sand	-	0	-0,5606	***
~~ Clay	~~Skeleton	-	0	0,6447	***
~~C	~~N	-	0	0,7161	***
sqrt(Bareground)	Slope	0.0174	0,0264	0,3021	*
DryMatter	Sand	0.0253	0,0018	0,3692	**
DryMatter	C	0.5268	0,0086	-0,2762	**
DryMatter	Skeleton	0.0219	0	-0,4583	***
DryMatter	sqrt(Bareground)	0.0744	0,0181	0,1761	*
Hardness	Skeleton	0.0118	0,0202	0,3723	*
Hardness	Clay	0.06	0,0272	-0,38	*
Soil_temp	Surface_temp	0.0253	0	0,6549	***
Plot.Code	sqrt(Bareground)	0.0243	0	0,694	***
Plot.Code	Skeleton	0.0051	0,028	-0,2033	*
Plot.Code	Surface_temp	0.0101	0,0019	0,2949	**

Table S 4: Overview of the estimated total nest number, the total area covered by each aggregation (m²), and the proportion of HND-area per year (%) per study site

	**Total nest number**			**Total area (m²)**			**HND-area (%)**		
**Site ID**	2022	2023	2024	2022	2023	2024	2022	2023	2024
1	6,926	4,221	6,808	396	374	261	12.46	19.07	28.07
2	7,958	12,515	7,955	146	139	131	60.28	80.50	58.17
3	6,708	9,090	230	300	344	51	12.75	32.74	7.55
4	16,342	28,742	35,231	446	462	432	40.67	59.32	71.91
5	3,360	6,757	5,298	71	76	70	39.69	59.44	63.94
6	71,482	88,352	98,942	906	978	846	41.09	55.77	65.66
7	2,713	4,493	1,110	108	172	70	44.84	31.01	27.40
8	3,659	5,364	2,975	105	184	134	39.06	24.31	12.00
9	4,162	6,054	5,890	83	96	77	59.08	49.70	62.40
10	19,802	16,421	42,789	823	466	662	33.82	11.57	58.87
11	2,576	645	851	101	48	79	40.78	30.66	16.75
12	20,355	31,643	8,642	357	469	213	27.46	48.62	32.20
13	8,254	15,113	9,093	167	226	344	26.28	53.60	40.61
14	5,337	9,951	12,204	259	301	247	22.76	38.31	39.76
15	6,702	4,035	1,862	182	215	110	24.40	18.24	9.50
16	6,979	7,308	3,212	539	474	357	49.48	25.68	10.97
17	1,313	1,982	1,235	115	144	77	44.57	28.12	31.54
18	1,902	3,124	1,408	149	107	34	19.99	34.28	11.21
19	29,815	51,509	23,397	666	728	644	45.58	51.91	23.73
20	12,609	9,681	11,220	188	263	214	61.69	33.94	44.76
21	785	636	1,337	105	75	69	43.43	14.14	29.80
22	9,794	13,175	6,836	249	380	185	23.85	36.97	52.95
23	2,888	2,474	3,746	145	100	146	19.36	29.81	33.31
24	13,939	22,748	2,907	237	401	217	46.93	40.10	17.46
25	19,186	29,538	32,414	197	248	274	54.30	44.23	69.57
26	10,386	16,601	7,559	160	197	189	31.35	58.28	49.22
27	1,623	1,1267	5,153	64	161	243	34.04	67.76	36.62
**Sum**	297,580	417,287	340,303						

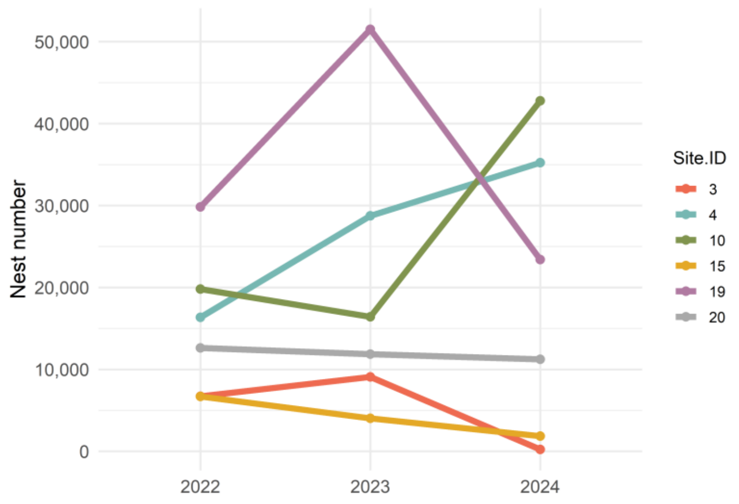

Figure S 8: Size development of selected nesting sites over three years: While some aggregations continuously grew (e.g., site 4), some nest numbers stayed constant (e.g., site 20) or declined (e.g., site 3 & 15). Most aggregations showed fluctuations (e.g., site 10 & 19).

Table S 5: Maximum nest densities (per m²), with two counts per year and nesting site

	2022		2023		2024	
**Site ID**	High 1	High 2	High 1	High 2	High 1	High 2
1	66	60	55	78	122	103
2	109	99	178	160	178	119
3	54	41	108	91	12	20
4	112	62	146	147	145	180
5	107	98	220	196	139	162
6	81	108	99	164	142	139
7	36	73	109	117	53	65
8	84	83	141	158	114	122
9	122	89	180	209	163	165
10	40	42	65	71	106	80
11	79	52	77	41	61	50
12	143	155	187	196	163	136
13	128	108	181	179	71	76
14	64	60	187	55	133	158
15	106	93	104	87	67	77
16	29	25	40	88	40	60
17	25	22	75	45	72	48
18	32	35	92	54	111	32
19	112	111	212	183	168	137
20	117	116	140	127	163	144
21	15	21	64	43	66	76
22	127	90	144	99	128	64
23	48	67	125	78	92	85
24	91	134	91	134	66	72
25	106	141	280	199	197	156
26	134	93	234	189	91	111
27	99	46	175	122	96	44

Table S 6: Overview of the slope (%) and the exposition of the slope within the HND-area of the aggregations.
** Site 32 also had HND-areas with an eastern and western exposition.*

	**Slope (%)**		
**Site ID**	2022	2023	**Exposition**
1	10.00	1.88	South-East
2	23.75	23.13	South-East
3	1.25	0.63	NA
4	1.25	0	NA
5	5.00	3.75	South-East
6	1.25	0	NA
7	1.25	0.63	NA
8	13.13	56.88	South-West
9	6.25	0	South
10	9.38	17.50	South-East
11	56.25	38.75	West
12	40.00	50.00	South-East
13	15.00	20.00	South-East*
14	8.75	6.25	South
15	0	0.63	NA
16	21.25	16.25	South-East
17	63.75	51.25	West
18	16.88	22.50	South-West
19	20.00	10.63	South-East
20	1.25	9.38	South-East
21	1.25	0	NA
22	26.88	8.75	South
23	5.63	3.75	South
24	1.25	3.75	South
25	6.25	8.13	South-East
26	30.00	8.75	South-West
27	18.13	20.63	South-West

Table S 7: Mean vegetation characteristics within the nesting sites from both plots within HND- and LND-areas, respectively, estimated in April 2022 and 2023 (%; mean of both years) and in March 2023 (%).

		**April 2022 & 2023**				**March 2023**			
**Site. ID**	**Type**	Vegetation	Bare ground	Moss	Litter	Vegetation	Bare ground	Moss	Litter
1	HND	42.50	41.25	3.80	12.50	45.00	42.50	0.10	12.50
1	LND	68.00	16.50	5.03	10.50	52.50	27.50	0.05	20
2	HND	58.50	36.00	0	5.50	62.00	37.00	0	1.00
2	LND	63.75	26.25	0.03	10.25	67.50	27.50	0.05	5.50
3	HND	59.75	33.75	0	6.53	62.50	30	0	7.55
3	LND	85.75	10	0	4.28	87.50	5.00	0	7.55
4	HND	56.00	27.75	10	6.28	62.00	20.50	15.00	2.55
4	LND	58.75	10.03	23.78	7.53	47.50	10.05	37.55	5.05
5	HND	22.50	69.75	1.28	6.53	12.50	84.50	2.55	0.55
5	LND	70.75	12.50	11.75	5.03	69.50	5.50	22.50	2.55
6	HND	67.00	26.25	0.25	6.53	76.50	22.50	0.50	0.55
6	LND	63.25	10.28	18.53	8.03	47.50	0.55	37.05	15.05
7	HND	64.75	30	0.25	5.00	77.00	20	0	3.00
7	LND	79.50	11.25	0.25	9.00	76.50	17.50	0.50	5.50
8	HND	21.25	43.50	27.50	7.75	25.00	19.50	50	5.50
8	LND	56.25	16.00	0.30	27.50	52.50	17.50	0.10	30
9	HND	74.50	21.75	0	3.80	99.00	1.00	0	0.10
9	LND	71.25	8.78	16.25	3.78	55.00	10.05	32.50	2.55
10	HND	89.25	10.25	0.50	0	93.50	5.50	1.00	0
10	LND	90	8.75	1.28	0	85.00	12.50	2.55	0
11	HND	42.25	41.00	0.28	16.50	52.00	32.00	0.55	15.50
11	LND	50	28.50	0.28	21.25	47.50	24.50	0.55	27.50
12	HND	48.75	46.00	0	5.25	67.50	22.50	0	10
12	LND	75.75	18.78	2.50	3.03	94.50	2.55	2.50	0.55
13	HND	62.50	30	0.03	7.50	72.50	22.50	0.05	5.00
13	LND	70.75	13.75	0.25	15.25	64.50	12.50	0.50	22.50
14	HND	55.00	44.75	0	0.53	77.50	22.50	0	0.55
14	LND	84.00	15.00	0.25	0.78	91.50	7.50	0.50	0.55
15	HND	87.00	12.50	0	0.55	95.00	5.00	0	0.10
15	LND	76.75	21.50	0	1.75	81.50	15.50	0	3.00
16	HND	62.25	30	0.03	7.75	67.00	30	0.05	3.00
16	LND	78.50	13.75	1.50	6.25	77.00	12.50	3.00	7.50
17	HND	11.25	86.00	0	2.78	7.50	92.00	0	0.55
17	LND	41.25	45.00	0.03	13.75	45.00	40	0.05	15.00
18	HND	37.50	56.00	0	6.53	35.00	64.50	0	0.55
18	LND	73.75	16.25	0	10	75.00	17.50	0	7.50
19	HND	45.00	48.75	0	6.30	47.50	52.50	0	0.10
19	LND	75.50	16.25	0.75	7.50	77.00	15.00	0.50	7.50
20	HND	52.50	42.25	0.03	7.75	40	57.00	0.05	3.00
20	LND	77.50	12.50	0.03	10	72.50	12.50	0.05	15.00
21	HND	24.00	68.25	0	7.75	33.00	64.00	0	3.00
21	LND	52.50	43.25	0	4.25	30.50	66.50	0	3.00
22	HND	53.75	41.25	0	5.00	47.50	45.00	0	7.50

Site. ID	Type	Vegetation	Bare ground	Moss	Litter	Vegetation	Bare ground	Moss	Litter
2	LND	82.00	9.00	0	9.00	94.00	3.00	0	3.00
23	HND	72.50	20	0	7.50	90	5.00	0	5.00
23	LND	83.50	7.75	0	8.75	89.50	3.00	0	7.50
24	HND	65.00	34.50	0	0.55	70	30	0	0.10
24	LND	76.00	16.25	1.25	6.50	65.00	25.00	2.50	7.50
25	HND	68.50	26.25	0.28	5.05	82.50	17.50	0.05	0.10
25	LND	68.50	10.25	18.75	2.55	79.50	3.00	17.50	0.10
26	HND	34.75	61.25	0	4.00	42.00	55.00	0	3.00
26	LND	68.50	18.75	4.00	8.75	69.50	15.00	8.00	7.50
27	HND	64.50	31.00	0.25	4.25	86.50	10	0.50	3.00
27	LND	74.25	19.00	0.50	6.30	91.00	8.00	1.00	0.10

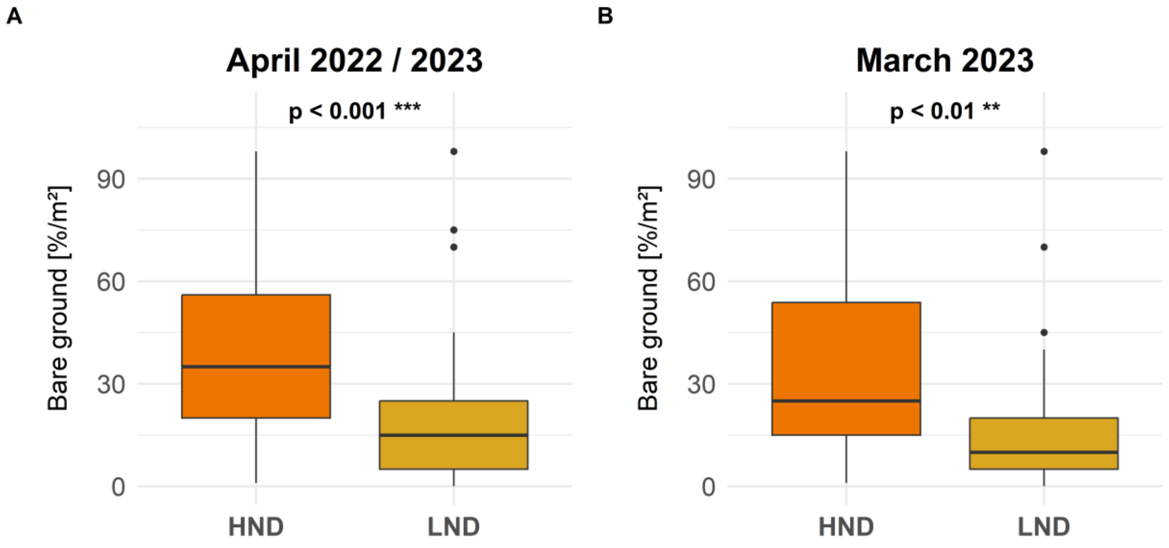

Figure S 9: Comparison between the proportion of bare ground (%/m²) in HND- and LND-areas, A) in April 2022 & 2023 during the bees' activity, and B) in March 2023 before the bees' activity.

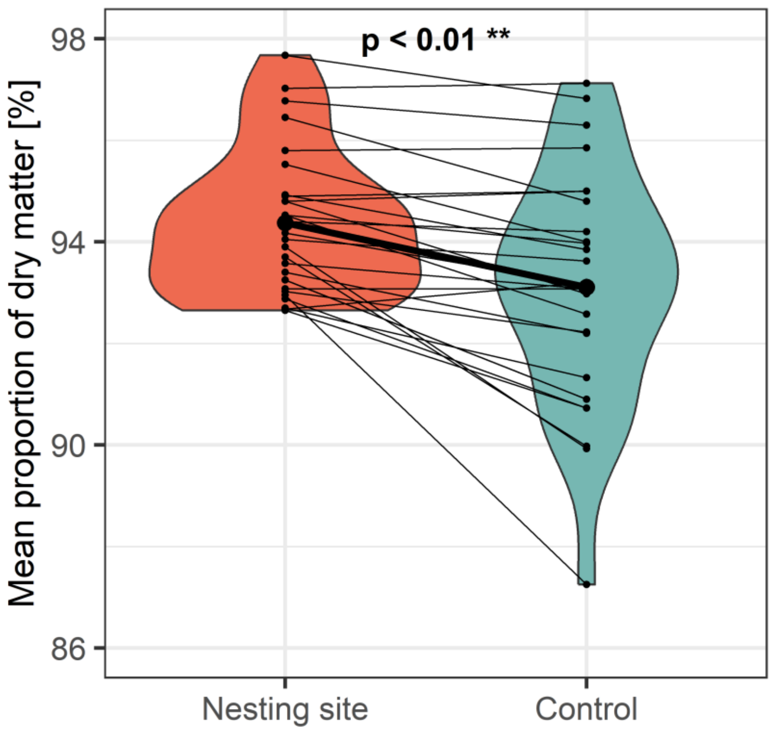

Figure S 10: Violin plot showing the comparison of dry matter proportions between nesting sites and controls. Dots show the mean values over all fours depth per study site, bold lines represent mean values over all study sites. Nesting sites showed significantly higher proportions of dry matter, particularly within 0-15 cm (see Fig. 5).

Table S 8: Canopy cover of the whole nesting site, the HND-area and LND-area (%).

	**Canopy cover (%)**			**HND-area under tree (%)**			**LND-area under tree (%)**		
**Site ID**	2022	2023	2024	2022	2023	2024	2022	2023	2024
1	49.74	55.73	51.05	27.76	77.32	46.19	52.86	50.64	52.95
2	79.17	75.90	72.12	74.80	73.79	69.82	85.79	84.60	75.30
3	23.34	21.93	65.71	33.17	13.18	93.04	21.91	26.19	63.48
4	23.24	29.99	31.01	13.22	11.83	32.39	30.11	56.48	27.46
5	100	100	100	100	100	100	100	100	100
6	52.83	49.70	55.94	37.41	50.86	62.85	63.58	48.24	42.73
7	82.59	58.36	77.53	67.32	80.89	100	95.00	48.24	69.05
8	67.67	52.16	64.62	63.21	52.66	59.75	70.52	52.00	65.29
9	59.82	39.99	54.16	77.28	64.88	61.41	34.61	15.39	42.13
10	0	0	0	0	0	0	0	0	0
11	100	100	100	100	100	100	100	100	100
12	21.25	24.23	35.44	32.74	25.54	65.43	16.90	22.98	21.19
13	34.65	32.49	32.27	59.94	34.65	42.26	25.64	30.01	25.43
14	66.34	70.04	68.67	73.40	65.19	68.15	64.26	73.05	69.01
15	0	0	0	0	0	0	0	0	0
16	26.25	32.93	36.75	36.76	56.11	70.78	15.94	24.91	32.55
17	93.03	78.60	74.94	92.98	90.67	87.97	93.07	73.88	68.93
18	76.74	92.46	100	100	100	100	70.93	88.53	100.00
19	96.75	89.74	89.63	95.86	91.61	86.50	97.50	87.73	90.61
20	80.72	80.63	82.35	79.36	82.76	73.32	82.91	79.54	89.67
21	57.62	64.50	69.11	97.04	66.20	81.50	27.35	64.22	63.85
22	57.68	60.52	55.10	46.40	80.02	46.74	61.21	49.08	64.52
23	96.00	96.52	97.63	99.88	100	100	95.06	95.04	96.45
24	64.26	40.82	46.99	57.81	57.77	60.60	69.96	29.48	44.11
25	34.41	64.26	48.96	40.28	83.41	55.01	27.44	49.07	35.11
26	39.71	45.70	48.42	16.89	36.90	49.94	50.13	58.01	46.94
27	0	0	0	0	0	0	0	0	0

Table S 9: Tree species found adjacent to the nesting sites, and their diameter in breast height (DBH). DBH was only measured when the tree consisted of a single trunk. On study site 11 no trees were found in the near surrounding.

Site.ID	Tree ID	Tree species	DBH
1	1.1	*Quercus robur*	1.11
1	1.2	*Acer rubrum*	0.25
1	1.3	*Platanus × hispanica*	0.81
1	1.4	*Quercus robur*	0.62
2	2.1	*Prunus padus*	0.27
2	2.2	*Tilia cordata*	0.44
2	2.3	*Carpinus betulus*	0.25
2	2.4	*Carpinus betulus*	0.21
2	2.5	*Tilia cordata*	0.38
2	2.6	*Carpinus betulus*	0.25
2	2.7	*Carpinus betulus*	0.30
2	2.8	*Prunus padus*	0.38
2	2.9	*Carpinus betulus*	0.22
2	2.10	*Carpinus betulus*	0.25
2	2.11	*Carpinus betulus*	0.19
3	3.1	*Corylus colurna*	0.30
3	3.2	*Corylus colurna*	0.29
3	3.3	*Corylus colurna*	0.29
3	3.4	*Corylus colurna*	0.25
3	3.5	*Corylus colurna*	0.25
4	4.1	*Tilia cordata*	0.59
4	4.2	*Acer platanoides*	0.08
4	4.3	*Acer platanoides*	0.07
4	4.4	*Betula pendula*	0.33
4	4.5	*Acer platanoides*	0.65
4	4.6	*Tilia cordata*	0.65
4	4.7	*Abies nordmanniana*	0.16
5	5.1	*Quercus rubra*	0.29
5	5.2	*Quercus rubra*	0.67
5	5.3	*Quercus rubra*	0.46
6	6.1	*Tilia cordata*	0.53
6	6.2	*Tilia cordata*	0.49
6	6.3	*Tilia cordata*	0.54
6	6.4	*Tilia cordata*	0.49
6	6.5	*Tilia cordata*	0.53
6	6.6	*Tilia cordata*	0.24
7	7.1	*Taxus baccata*	0.08
7	7.2	*Taxus baccata*	0.10
7	7.3	*Quercus robur*	0.37
7	7.4	*Quercus robur*	0.73
7	7.5	*Quercus robur*	0.24
7	7.6	*Betula pendula*	0.35
7	7.7	*Quercus robur*	0.37
7	7.8	*Betula pendula*	0.38
7	7.9	*Betula pendula*	0.33

Site.ID	Tree ID	Tree species	DBH
8	8.1	*Quercus robur*	0.22
8	8.2	*Quercus rubra*	0.48
8	8.3	*Quercus robur*	0.38
8	8.4	*Quercus rubra*	0.32
8	8.5	*Quercus robur*	0.25
8	8.6	*Quercus robur*	0.19
8	8.7	*Quercus robur*	0.19
9	9.1	*Aesculus hippocastanum*	0.32
9	9.2	*Tilia cordata*	0.53
11	11.1	*Quercus robur*	0.22
11	11.2	*Quercus robur*	0.27
11	11.3	*Quercus robur*	0.32
11	11.4	*Quercus robur*	0.41
11	11.5	*Quercus robur*	0.65
12	12.1	*Picea abies*	0.37
12	12.2	*Prunus avium*	0.59
12	12.3	*Tilia cordata*	0.38
12	12.4	*Malus domestics*	0.07
12	12.5	*Tilia cordata*	0.38
12	12.6	*Acer campestre*	NA
12	12.7	*Tilia cordata*	0.40
12	12.8	*Malus domestics*	0.05
12	12.9	*Malus domestics*	0.03
12	12.10	*Acer campestre*	NA
13	13.1	*Quercus robur*	0.43
13	13.2	*Aesculus hippocastanum*	0.41
13	13.3	*Aesculus hippocastanum*	0.25
13	13.4	*Aesculus hippocastanum*	0.29
13	13.5	*Quercus robur*	0.48
13	13.6	*Aesculus hippocastanum*	0.29
14	14.1	*Quercus robur*	0.84
15	15.1	*Shrubs (Corylus avellana, Carpinus betulus, Prunus padus, Cornus sanguinea)*	NA
15	15.2	*Quercus robur*	0.21
15	15.3	*Quercus robur*	0.22
16	16.1	*Tilia cordata*	0.30
16	16.2	*Acer platanoides*	NA
16	16.3	*Acer platanoides*	0.46
16	16.4	*Acer platanoides*	0.92
17	17.1	*Acer platanoides*	0.37
17	17.2	*Acer platanoides*	0.21
17	17.3	*Acer platanoides*	0.30
17	17.4	*Acer platanoides*	0.27
17	17.5	*Acer platanoides*	0.14
17	17.6	*Acer platanoides*	0.32
18	18.1	*Tilia cordata*	0.62
18	18.2	*Fraxinus excelsior*	0.62
19	19.1	*Quercus robur*	0.95

Site.ID	Tree ID	Tree species	DBH
19	19.2	*Quercus robur*	0.92
19	19.3	*Aesculus hippocastanum*	0.94
19	19.4	*Aesculus hippocastanum*	0.75
19	19.5	*Tilia cordata*	0.78
19	19.6	*Quercus robur*	0.16
19	19.7	*Quercus robur*	0.73
19	19.8	*Quercus robur*	0.69
20	20.1	*Tilia cordata*	0.29
20	20.2	*Tilia cordata*	0.72
20	20.3	*Tilia cordata*	0.51
20	20.4	*Tilia cordata*	0.91
20	20.5	*Tilia cordata*	0.91
21	21.1	*Populus tremula*	0.94
21	21.2	*Populus tremula*	0.70
21	21.3	*Populus tremula*	0.73
21	21.4	*Populus tremula*	1.07
21	21.5	*Acer campestre*	0.10
21	21.6	*Acer campestre*	0.10
21	21.7	*Platanus × hispanica*	0.83
21	21.8	*Aesculus hippocastanum*	0.27
22	22.1	*Acer platanoides*	0.70
22	22.2	*Acer platanoides*	0.54
22	22.3	*Tilia cordata*	0.70
22	22.4	*Tilia cordata*	0.62
22	22.5	*Tilia cordata*	0.53
22	22.6	*Tilia cordata*	0.45
23	23.1	*Aesculus hippocastanum*	0.47
23	23.2	*Aesculus hippocastanum*	0.43
23	23.3	*Aesculus hippocastanum*	0.34
23	23.4	*Acer pseudoplatanus*	0.80
23	23.5	*Acer pseudoplatanus*	0.76
24	24.1	*Tilia cordata*	0.80
24	24.2	*Tilia cordata*	0.80
24	24.3	*Quercus robur*	0.45
24	24.4	*Abies nordmanniana*	0.14
25	25.1	*Betula pendula*	0.64
25	25.2	*Tilia cordata*	0.25
26	26.1 - 25.5	*Populus nigra*	0.48
26	26.6	*Celastrus orbiculatus*	0.08
26	26.7	*Celastrus orbiculatus*	0.29
26	26.8	*Quercus robur*	0.32
26	26.9	*Quercus robur*	0.32
26	26.10	*Quercus robur*	0.32
26	26.11	*Quercus robur*	0.13
27	27.1	*Fraxinus excelsior*	0.05

*Table S 10: Overview of the total nest number per tumuli category (*A. vaga*, smaller or larger bee species), summed up from both rounds, and the mean vegetation characteristics (%) per study site and plot type ('control', 'sparse' & 'bare').*

		**Total nest number**			**Vegetation characteristics (%)**			
**Site ID**	**Plot**	*A. vaga*	Smaller	Larger	Vegetation	Bare ground	Moss	Litter
3	Control	0	3	0	64.50	35.00	1.00	0.10
3	Sparse	1	0	0	57.50	42.50	0.05	0.10
3	Bare	0	4	8	8.00	92.00	0.05	0.10
5	Control	0	0	1	44.00	0.10	47.50	8.50
5	Sparse	0	56	1	9.50	79.50	10.00	1.00
5	Bare	1	70	0	0	99.00	0.10	0.55
6	Control	0	0	0	99.00	0.10	0.05	0.55
6	Sparse	0	0	0	90.00	10.00	0.05	0.05
6	Bare	0	5	6	5.00	94.50	0.00	0.05
11	Control	0	0	0	64.50	15.00	5.50	15.00
11	Sparse	1	0	1	36.50	55.00	3.00	5.50
11	Bare	4	6	1	0	99.45	0.10	0.55
13	Control	1	81	0	51.00	48.50	0.05	0.55
13	Sparse	12	221	2	70.00	30.00	0.05	0.05
13	Bare	0	241	0	8.00	92.00	0.05	0.10
16	Control	0	6	1	98.50	1.00	0.55	0.05
16	Sparse	0	11	0	85.00	15.00	0.05	0.10
16	Bare	0	108	0	8.00	92.00	0.10	0.10
17	Control	0	2	0	52.50	20.00	0.10	27.50
17	Sparse	0	1	1	57.50	32.50	0.05	10.00
17	Bare	0	2	0	0.50	92.00	0.05	7.50
18	Control	1	42	0	91.00	8.50	0.05	0.55
18	Sparse	0	6	1	82.50	17.50	0.05	0.05
18	Bare	4	163	3	3.00	97.00	0.10	0.10
19	Control	0	0	0	41.50	0	57.50	1.00
19	Sparse	0	2	2	66.00	30.00	3.00	1.00
19	Bare	0	3	1	7.50	91.50	0.55	0.55
21	Control	0	1	0	65.00	25.00	0.10	10.00
21	Sparse	0	0	3	60.00	30.00	0.00	10.00
21	Bare	0	0	5	1.00	89.00	0.00	10.00
22	Control	0	11	1	97.00	3.00	0.10	0.10
22	Sparse	0	6	1	94.50	5.50	0.10	0.05
22	Bare	0	248	3	8.00	92.00	0.00	0.05
23	Control	1	31	2	81.00	17.50	1.00	0.55
23	Sparse	0	21	10	54.50	45.00	0.10	0.10
23	Bare	0	47	4	7.50	92.00	0	0.55
26	Control	1	1	2	60.00	22.50	0.10	17.50
26	Sparse	1	2	0	47.50	32.50	0.05	20.00
26	Bare	1	2	3	17.50	62.50	0.05	20.00

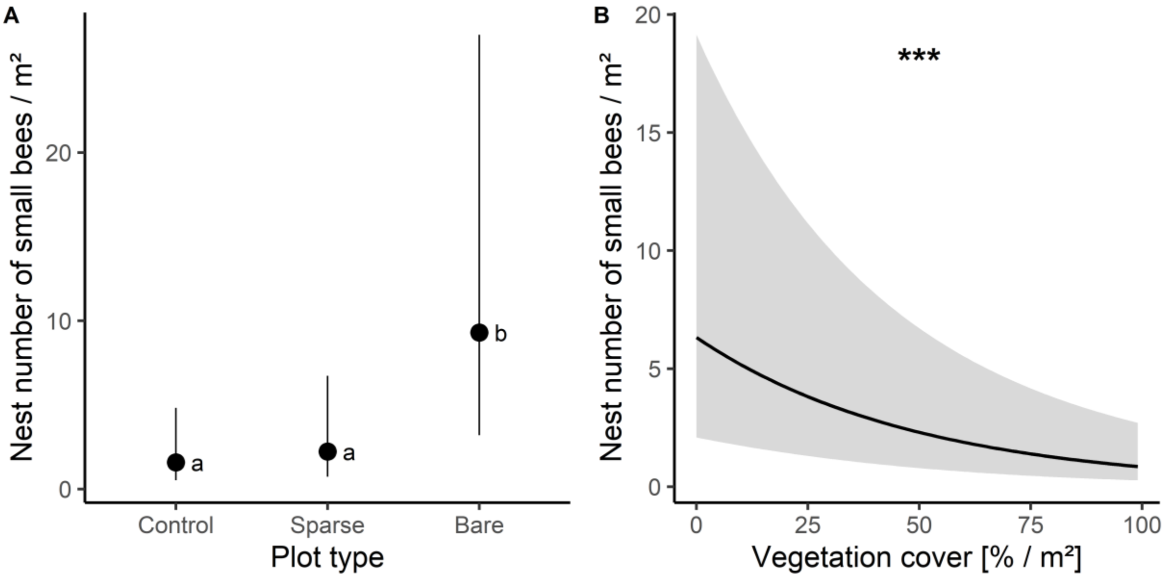

Figure S 11: Effect of A) the plot type ('control', 'sparse' & 'bare'), and B) the vegetation cover (%/m²) on the nest number of small bee species per m².

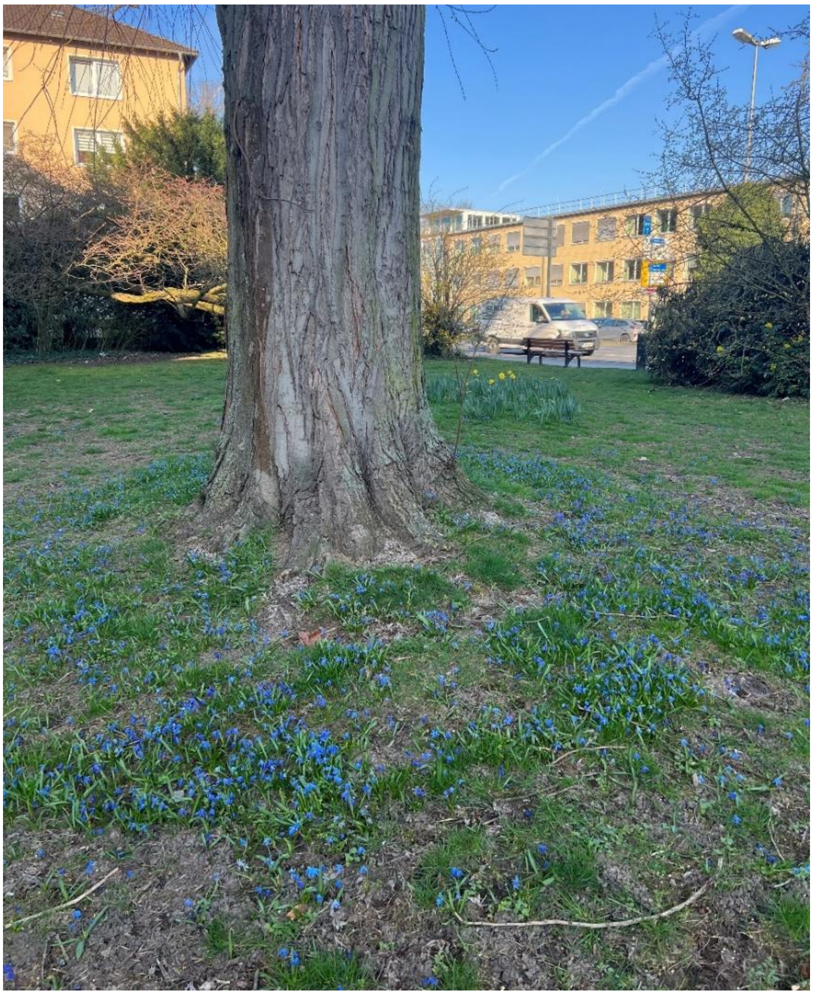

Figure S 12: Ephemeral spring flowers growing under single trees within parks

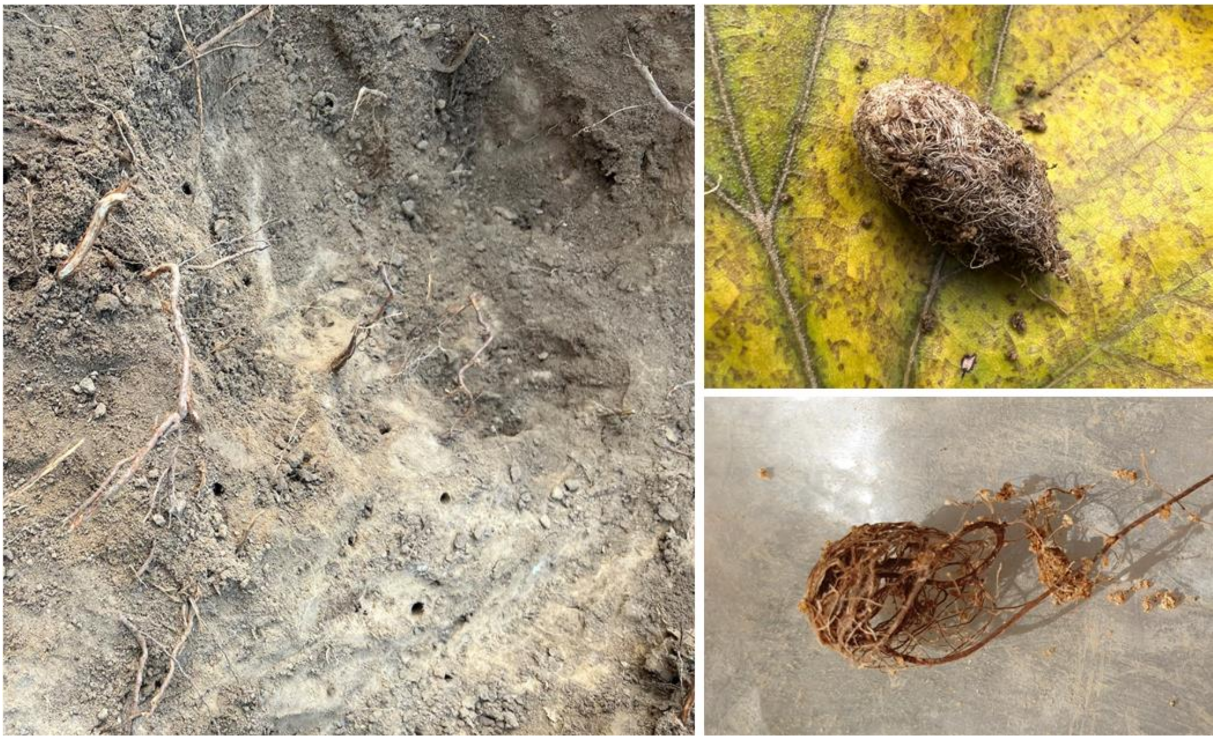

Figure S 13: Nest tunnels of A. vaga *within the root system of a tree, and roots occasionally found within brood cells of* A. vaga*.*

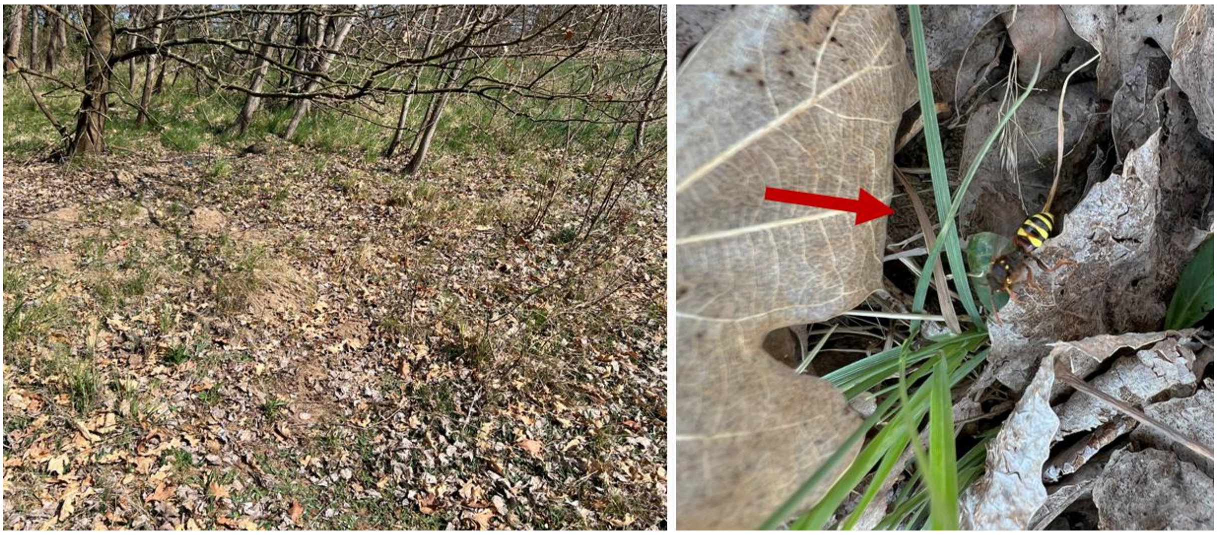

Figure S 14: Left side: Nesting site of A. vaga *with a dense leaf litter cover.*
Right side: A. vaga *nest hiden under leaves, with its cuckoo bee,* Nomada lathburiana*.*
This nesting site was not included in the study, as it was only recently discovered in 2025.

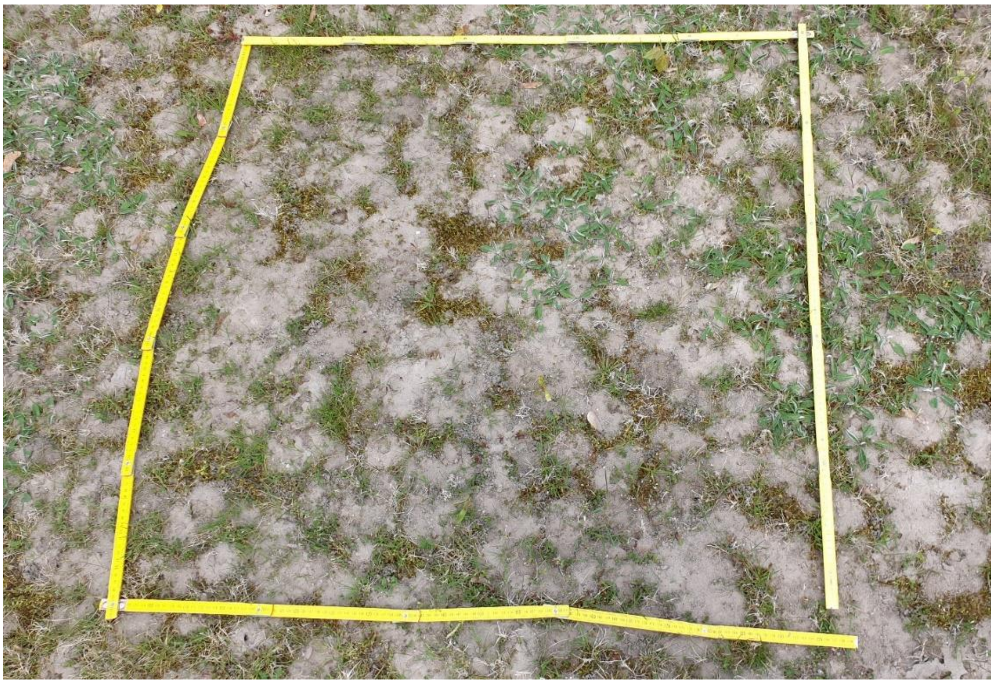

Figure S 15: High nest densities within areas with high proportions of moss

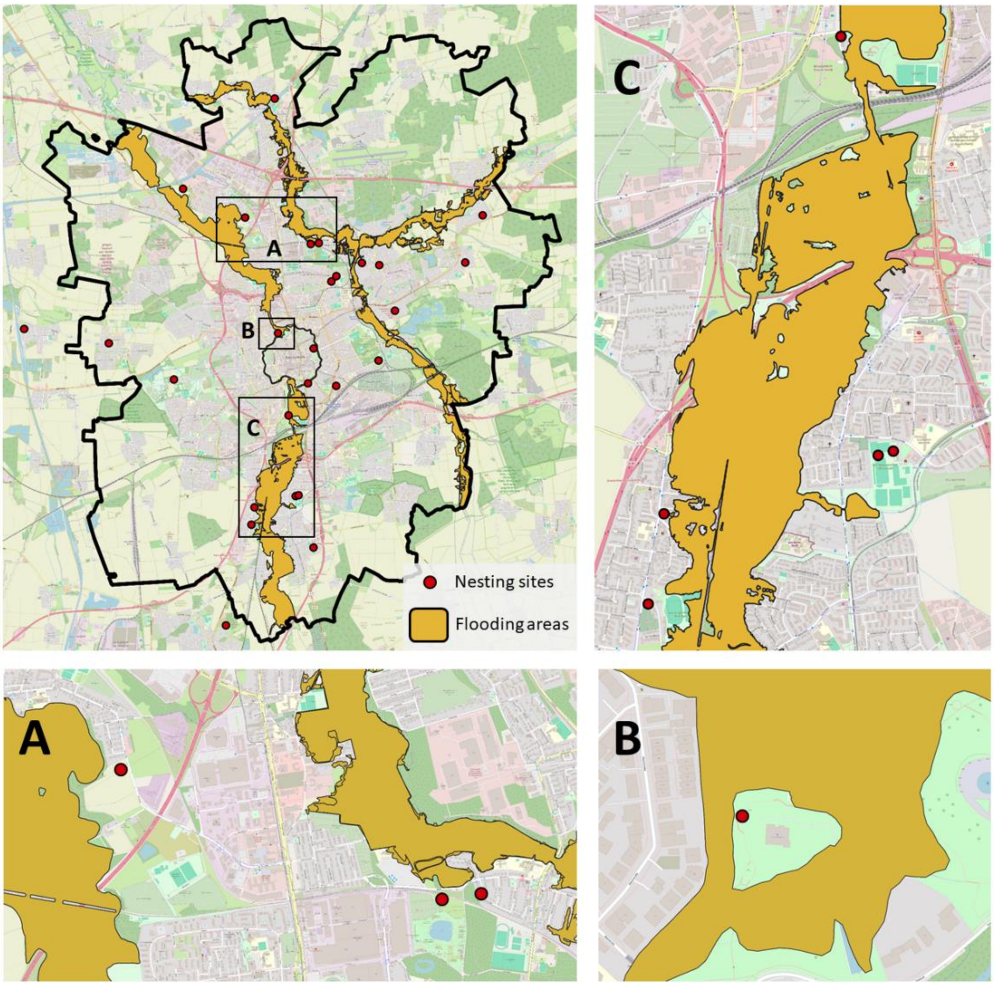

Source: OSM, author: H. Gardein (JKI), 2025

Figure S 16: Location of the studied nesting sites in relation to statutorily defined flooding areas

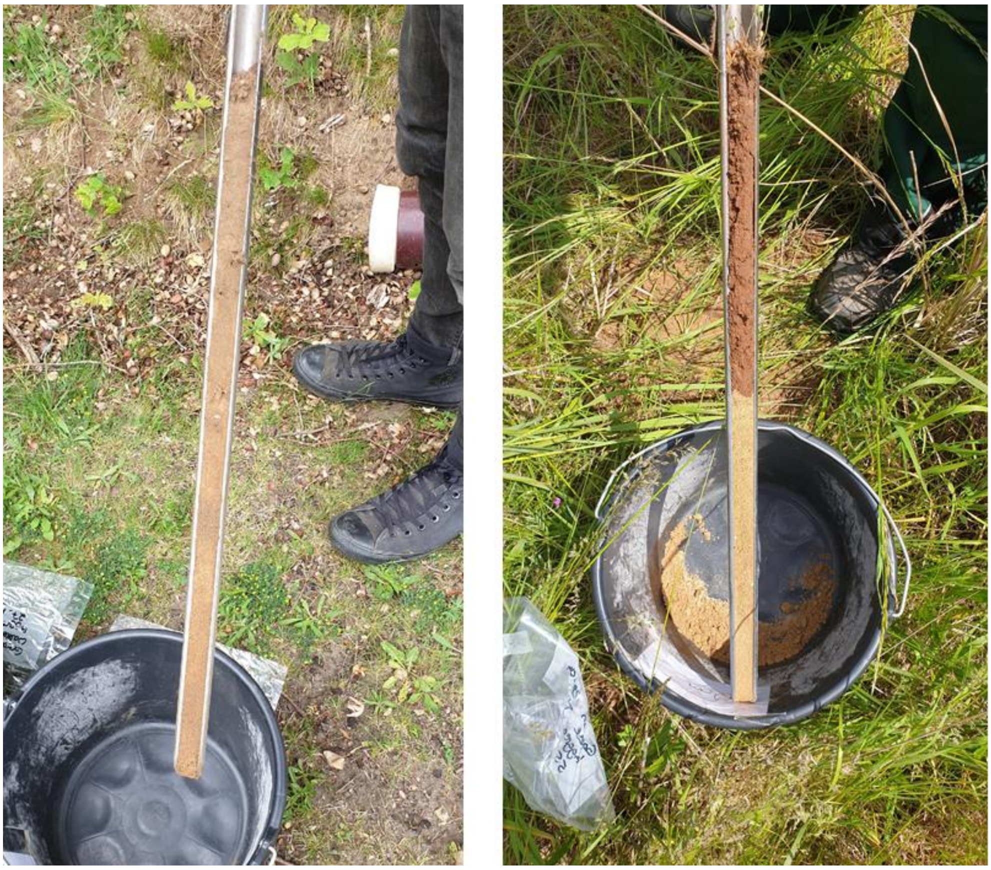

Figure S 17: Examples of soil samples. Left picture: A homogeneous sample of study site 11 with high sand proportions (0-15 cm: 91.72%, 15-30 cm: 91.28%, 30-45 cm: 92.8%, 45-60 cm: 93.68%). Right picture: Study site 27 with a low sand proportion in the upper horizont (0-15 cm: 57.84%, 15-30 cm: 69.48%, 30-45 cm: 84.76%, 45-60 cm: 88.44%)

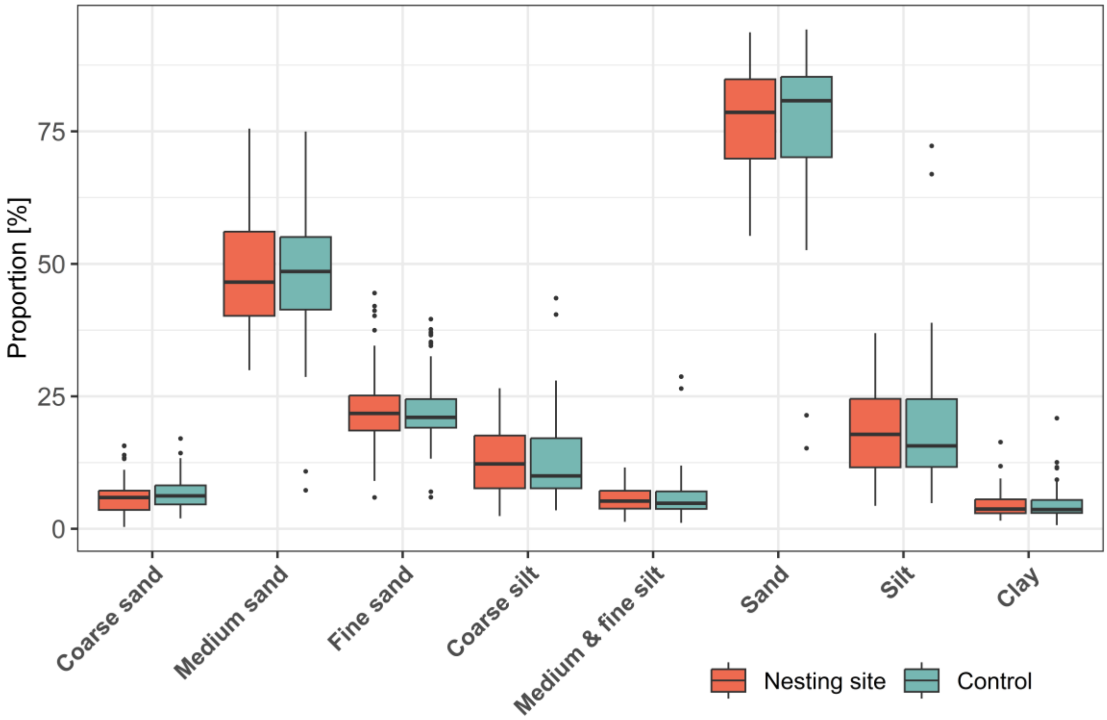

Figure S 18: Boxplots of the soil texture subclassifications within the nesting sites and control plots. Detailed information on proportions of subclasses can be found in the associated data repository (Gardein et al., 2025)

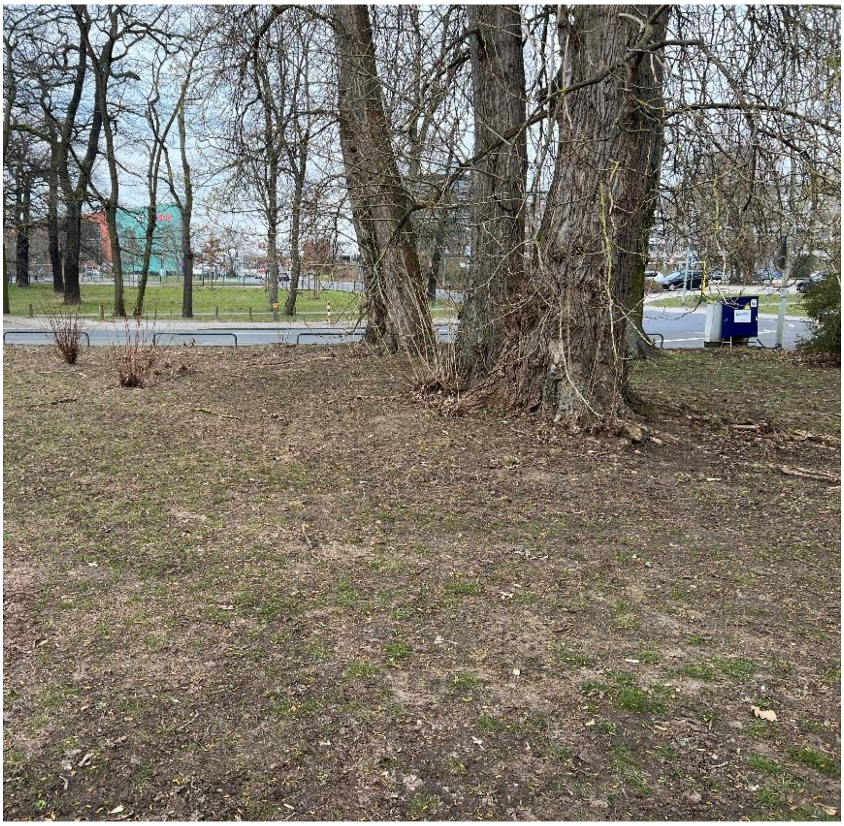

Figure S 19: Nesting site 21 on a flat ground, with high proportions of bare ground

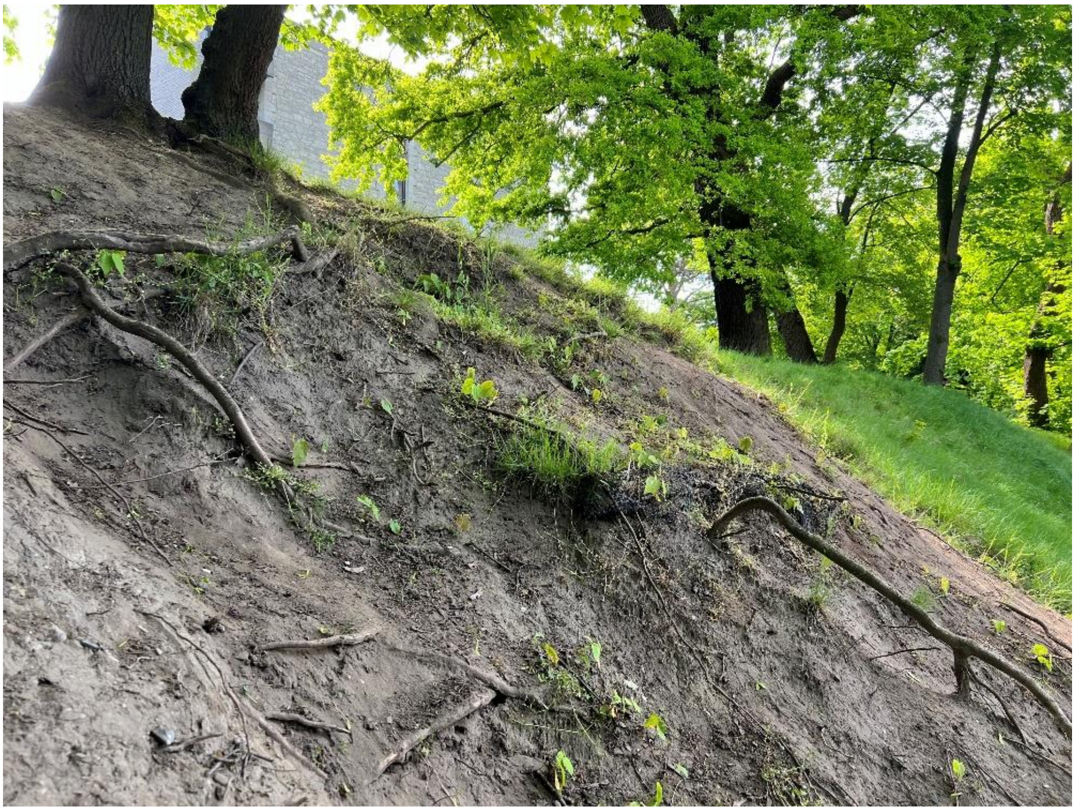

Figure S 20: Nesting site 17 on a steep slope facing west, with a high proportion of bare ground

Table S 11: Overview of the green space land use type, and the official soil type classification, based on the BÜK200 data (BGR, 2013), per nesting site. The mean penetration resistance ('soil hardness'; in MPa) across all 60 cm and all three penetrations is given, as well as the mean depth (in cm) until which the penetrations could be conducted (only for the nesting sites, not control areas).

Site ID	Green space type	Soil type classification	Additional information	Mean penetration resistence	Mean penetration depth
1	Cemetery	Cambisol		3.15	36.00
2	Playground	Brown Podzolic Soil		2.13	60.00
3	Roadside verge	Brown Podzolic Soil	Anthropogen. modified soil	3.39	60.00
4	Private front yard	Anthrosol	Anthropogen. modified soil	2.16	38.33
5	Institution premises	Cambisol	Anthropogen. modified soil	3.17	39.00
6	Institution premises	Gleyic Cambisol	Anthropogen. modified soil	2.08	55.00
7	Roadside verge	Gleyic Cambisol	Anthropogen. modified soil	2.24	34.00
8	Extensive grassland	Gleyic Cambisol	Anthropogen. modified soil	2.81	52.67
9	Private front yard	Gleyic Cambisol	Anthropogen. modified soil	2.48	60.00
10	Extensive grassland	Brown Podzolic Soil		3.38	60.00
11	Extensive grassland	Brown Podzolic Soil		1.60	29.33
12	Institution premises	Brown Podzolic Soil		2.83	60.00
13	Park	Stagnosol		2.51	44.67
14	Cemetery	Luvisol		2.64	54.67
15	Playground	Luvisol		2.22	42.33
16	Park	Anthropogenic fillings		1.81	60.00
17	Park	Anthropogenic fillings		1.25	60.00
18	Park	Anthropogenic fillings		2.05	58.33
19	Park	Anthropogenic fillings		1.37	60.00
20	Park	Cambisol	Anthropogen. modified soil	1.44	60.00
21	Park	Gleyic Cambisol		4.66	11.67
22	Sports field	Gleyic Cambisol		1.88	60.00
23	Playground	Gleyic Cambisol		2.87	49.33
24	Private front yard	Gleyic Cambisol		1.04	60.00

Site ID	Green space type	Soil type classification	Additional information	Mean penetration resistence	Mean penetration depth
25	Cemetery	Gleyic Cambisol		1.18	60.00
26	Roadside verge	Gleyic Cambisol		3.06	53.00
27	Extensive grassland	Luvisol		2.29	53.00

References

BGR (2013) *Bodenübersichtskarte 1:200.000 (BÜK200) - CC3926 Braunschweig*. Available from: www.numis.niedersachsen.de [Accessed 13 May 2025].

Schüller, H. (1969): Die CAL-Methode, eine neue Methode zur Bestimmung des pflanzenverfügbaren Phosphates in Böden. In: *J Plant Nutrition & Soil* 123 (1), S. 48–63. DOI: 10.1002/jpln.19691230106.

Gardein, H., Diekötter, T., Bloem, E. & Greil, H. (2025) Soil properties of nesting sites of the ground-nesting wild bee *Andrena vaga* and corresponding uncolonized control areas [Data repository]. *OpenAgrar Repository*. Available from: https://www.openagrar.de/receive/openagrar_mods_00107758.
